# One‐Pot Biocatalytic In Vivo Methylation‐Hydroamination of Bioderived Lignin Monomers to Generate a Key Precursor to L‐DOPA

**DOI:** 10.1002/ange.202112855

**Published:** 2022-01-10

**Authors:** James L. Galman, Fabio Parmeggiani, Lisa Seibt, William R. Birmingham, Nicholas J. Turner

**Affiliations:** ^1^ Department of Chemistry University of Manchester Manchester Institute of Biotechnology 131 Princess Street M1 7DN Manchester UK; ^2^ FabricNano 184–192 Drummond Street NW1 3HP London UK; ^3^ Department of Chemistry Materials and Chemical Engineering “G. Natta” Politecnico di Milano Piazza Leonardo Da Vinci 32 20131 Milano Italy

**Keywords:** Amino Acids, Biocatalysis, Circular Economy, Cofactor Regeneration, Protein Engineering

## Abstract

Electron‐rich phenolic substrates can be derived from the depolymerisation of lignin feedstocks. Direct biotransformations of the hydroxycinnamic acid monomers obtained can be exploited to produce high‐value chemicals, such as α‐amino acids, however the reaction is often hampered by the chemical autooxidation in alkaline or harsh reaction media. Regioselective *O*‐methyltransferases (OMTs) are ubiquitous enzymes in natural secondary metabolic pathways utilising an expensive co‐substrate *S*‐adenosyl‐l‐methionine (SAM) as the methylating reagent altering the physicochemical properties of the hydroxycinnamic acids. In this study, we engineered an OMT to accept a variety of electron‐rich phenolic substrates, modified a commercial *E. coli* strain BL21 (DE3) to regenerate SAM in vivo, and combined it with an engineered ammonia lyase to partake in a one‐pot, two whole cell enzyme cascade to produce the l‐DOPA precursor l‐veratrylglycine from lignin‐derived ferulic acid.

## Graphical abstract

Protein and strain engineering combined. The combination of two engineered enzymes (a methyltransferase and an ammonia lyase) and an engineered *E. coli* strain (for regeneration of the SAM cofactor) has been developed to enable a fully biocatalytic one‐pot methylation–hydroamination cascade. As an example, the synthesis of l ‐veratrylglycine from renewable lignin‐derived ferulic acid has been demonstrated, in high yield and excellent *ee*.

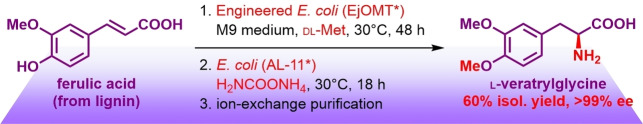

## Introduction

Lignin is by far the most abundant renewable source of aromatic compounds in Nature comprising 15–30 % of the dry weight of lignocellulose, a promising candidate feedstock from which to obtain a range of aromatic chemicals. There are growing incentives to replace crude oil and fossil raw materials with greener, and sustainable alternatives such as renewable biomass.^[1]^ One of the most common structural moieties present in plant lignin polymers is based on cinnamic acid derivatives bearing one or more electron‐donating groups.^[2]^ In particular, ferulic acid is one of the major hydroxycinnamic acids widely found in plant cell‐walls such as cereal bran (0.5 % w/w), corn kernel (1 g kg^−1^), maize bran (3.0 % w/w), sugar beet pulp (0.8 % w/w), and wheat brans (0.05 g kg^−1^).[^3a^, ^3b^, ^3c^] Therefore, there are valuable opportunities for recovering such chemicals from waste streams of agriculture and paper industries.

Depolymerisation of corn bran lignin has been demonstrated thermochemically via alkaline hydrolysis and high temperatures to obtain hydroxycinnamates (e.g., ferulic acid).^[4]^ The other attractive depolymerisation technique is to use grass lignins (e.g., sugar cane bagasse) as feedstocks to produce bioproducts from microbial degradation using fungal hosts, e.g. white‐rot fungi (*Phanerochaete chrysosporium*) which release extracellular lignin peroxidases and manganese‐dependent peroxidases.^[5]^ Bacterial degradation,^[6]^ although not as extensively studied as fungal strains, can depolymerise lignin in the bacterial phyla *Proteobacteria*, *Actinobacteria* and *Firmicutes* utilising oxidative laccases and heme peroxidases^[7]^ DyP under aerobic conditions. *Rhodococcus* in particular, can convert kraft lignin and wheat straw to aromatic dicarboxylic acids and other low‐molecular weight phenolic byproducts.^[8]^ Hydroxycinnamates obtained by chemical or biological processes can be further metabolised to higher‐value products such as flavours (e.g., vanillin^[9]^), fragrances (e.g., 4‐vinylphenol,^[10]^ coniferol^[11]^ and polymer precursors (e.g., styrene^[12]^). Biological production of these monomers is intrinsically sustainable and utilises green technologies, avoiding toxic (and expensive) metal catalysts, minimising waste and using less energy than conventional chemical methods.

The development of biocatalysts for further bioconversion of these phenolic acid derivatives from sugarcane bagasse to APIs (such as l‐DOPA to treat Parkinson's disease) provides a plant‐based platform for pharmaceuticals as an environmentally friendly alternative to petroleum‐based chemistry, thereby contributing towards a bio‐based economy.

**Scheme 1:**
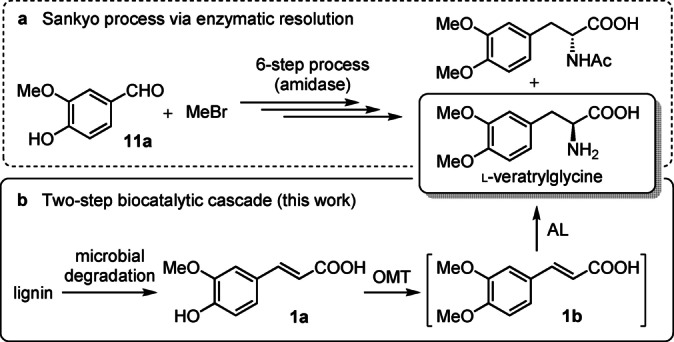
Synthetic approaches to l‐veratrylglycine. a) Sankyo process, requiring the use of an amidase for enzymatic chiral resolution. b) This work: a one‐pot, two‐step enzymatic process involving an *O*‐methyltransferase (OMT) and ammonia lyase (AL).

There are several examples in the literature of processes that exploit cell‐free and whole cell biocatalytic systems such as esterification, amination, decarboxylation, halogenation and C−C ring‐cleavage reactions on phenolic acids monomers derived from lignin.^[13]^ In order to diversify the current enzyme toolbox we investigated an unexplored enzyme cascade to generate l‐veratrylglycine, an important precursor in the production of L‐DOPA (Scheme 1). The current Sankyo process^[14]^ for l‐DOPA production requires multiple chemical protection/deprotection steps, handling of highly toxic substances such as MeBr, and an enzymatic chiral resolution with an amidase which cannot exceed the theoretical yield of 50 %.

Unfortunately, direct biotransformations of hydroxycinnamates (especially with ‐OH groups at the *ortho*/*para* position) is often problematic because of autoxidation in alkaline media via radical‐radical coupling mechanisms. This autoxidation can be mitigated by the addition of a radical scavenger^[15]^ or through the formation of reactive quinones, leading to dimeric and oligomeric products and in some cases the browning of solution.^[16]^ Direct hydroamination of hydroxycinnamates with an ammonia lyase for example, has proven challenging because of the high ammonia concentrations and high pH required to shift the equilibrium to product formation.[^17a^, ^17b^] Moreover, the substrates can also be decarboxylated by endogenous prenylated flavin‐containing ferulic acid decarboxylases^[18]^ in *E. coli* whole cells resulting in decreased product yields and the formation of undesirable side products.

Regioselective *O*‐methyltransferases (OMTs) are ubiquitous enzymes in natural secondary metabolic pathways catalysing the transfer of a methyl group from *S*‐adenosyl‐L‐methionine (SAM) in an S_N_2‐type reaction onto alcohols or phenols generating *S*‐adenosylhomocysteine (SAH) as a by‐product. Methylation reduces the number of reactive hydroxyl groups, altering the physicochemical characteristics (e.g., solubility, lipophilicity), odour/taste,^[19]^ chemical reactivity, or bioactive properties^[20]^ of the compounds. Extensive studies have been performed on eukaryotic catechol *O*‐methyltransferases (COMTs) where both human and rodent COMTs predominantly methylate ‐OH groups at the *meta*‐position of natural or synthetic catechols important in mammalian regulation and detoxification of neurotransmitters such as dopamine.[^21^, ^22^] There are few reports of OMTs that transfer the methyl group to the *para‐*position in substituted catechols: a recent report described engineering of an isoeugenol 4‐*O*‐methyltransferase IEMT from *Clarkia breweri* flowers by substituting key amino acid residues to resemble corresponding residues in an endogenous COMT, its purported evolutionary origin with which it shared 83 % protein sequence identity.^[23]^ Tang and co‐workers identified selectivity residues that switched substrate specificity between the related *O*‐methyltransferases, showing enhanced *para*‐regioselectivity towards 3,4‐dihydroxybenzoic acid. However, the enzyme was predominantly *meta*‐selective with other phenolic substrates screened.^[24]^ A catechol 4‐*O*‐methyltransferase (SafC) has been characterised from the biosynthesis of saframycin in *Myxococcus xanthus* and was demonstrated to have excellent *para*‐regioselectivity on a few catechols such as the physiological intermediate substrate l‐DOPA,^[25]^ and the phenolic acids dihydrocaffeic acid and 3,4‐dihydroxyphenylacetic acid but poor regioselectivity with caffeic acid.^[26]^

In this study, we investigated the substrate scope of an underexplored putative 4‐*O*‐methyltransferase from *Eriobotrya japonica* (EjOMT)^[27]^ and engineered the enzyme for excellent *para*‐regioselectivity. This OMT was previously known to have low relative activity with guaiacol‐type substrates and no activity with catechols. Methylation of the *para*‐OH group precludes autooxidation of hydroxycinnamates, and enabled the intermediate product to be stable under conditions for hydroamination with an ammonia lyase to yield L‐veratrylglycine.^[28]^ Moreover, as SAM is an unstable, expensive cofactor that is required in stoichiometric amounts for in vitro methylation reactions (typically making it an infeasible approach on an industrial scale), we addressed the cofactor limitations by introducing a SAM regeneration system in vivo by fine‐tuning genes beneficial to the methyl cycle pathway with the addition of only 2 equivalents of exogenous dl‐methionine.

## Results and Discussion

The codon optimised *O*‐methyltransferase gene from *Eriobotrya japonica* (EjOMT)^[27]^ was recombinantly expressed in *E. coli* with the addition of an *N*‐terminal His_6_‐tag. Previous biochemical studies had revealed the wild‐type (WT) enzyme gave 8 % product conversion for 1 mM ferulic acid **1** **a** at 25 °C for 15 minutes (compared to >99 % conversion with guaiacol). Despite the poor relative product conversion, purified recombinant EjOMT gave >99 % conversion of ferulic acid **1** **a** to **1** **b** within 18 h using 2 equiv of SAM disulfate tosylate. Other electron‐rich hydroxycinnamic acid derivatives which are relevant to lignocellulosic biomass degradation (e.g., *p*‐coumaric, caffeic and sinapinic acid) gave little or no conversion to the *para*‐methylated products. Our initial goal was to increase WT product conversion of *p*‐coumaric acid **2** **a** which gave **2** **b** at 16 % after 18 hours.

An energy‐minimised homology model was constructed (see Supporting Information), based on the structure of a coniferol‐9‐*O*‐methyltransferase LnCa9OMT from *Linum nodiflorum* (pdb: 4E70) which shares a 42 % protein sequence identity.^[29]^ Isolated co‐crystals with coniferol bound in the enzyme active site revealed that the *para*‐OH and *meta*‐OCH_3_ are stabilised by hydrogen bonding with a serine amino acid residue (S122). The corresponding position in EjOMT had evolved to a hydrophobic isoleucine residue (I133), therefore we hypothesized that restoring the serine residue would stabilise hydroxycinnamic acid substrates and increase activity for *p*‐coumaric acid **2** **a**. Indeed, the variant EjOMT I133S gave a 2.8‐fold increase in conversion (45 %) of **2** **a** to **2** **b**, while not compromising ferulic acid turnover.

Encouraged by our initial findings we performed an alanine‐valine scanning^[30]^ directed at conserved amino acid residues decorating the hydrophobic binding cavity, which sought to determine residues essential for stability and/or substrate binding. Five amino acids in total with non‐polar side chains interacting with a bound coniferol molecule in the enzyme active site were analysed (L129, M132, L138, F186 and L342). Mutations of L129, M132 and F186 to either A or V greatly reduced or abolished methyltransferase activity and were not considered further. The double mutant I133S/L138V afforded better results in comparison with the single mutant I133S and WT: even though conversion rates of **2** **a** to **2** **b** were similar, using sinapinic acid **3** **a** as a substrate afforded higher conversion to **3** **b** (97 %) than either I133S or WT (48 % and 45 %, respectively). It can be argued that increasing the size of the hydrophobic cavity increased substrate affinity, better accommodating the 5‐OCH_3_ group. Surprisingly, the incorporation of L342V or L342A mutations to the double mutant I133S/L138V both gave >99 % conversion of *p*‐coumaric acid **2** **a** to **2** **b**. Presumably, further opening of the hydrophobic cavity allows *p*‐coumaric acid to freely rotate to be accommodated in the binding pocket. Even more interestingly, the triple variant I133S/L138V/L342V was shown to have improved protein solubility and better activity against sinapinic acid **3** **a** (>99 % conversion to **3** **b**) compared to the I133S/L138V/L342A variant (24 %). The WT enzyme could not accept caffeic acid **4** **a** as a substrate but the single variant I133S gave 75 % yield of the dimethylated product **1** **b**, likely due to the presence of excess SAM used in the biotransformation, but the triple variant I133S/L138V/L342V was highly *para*‐regioselective (97 %) affording isoferulic acid **4** **b**, regardless of SAM concentration.

A more thorough investigation was performed, screening WT and all variants against a broad panel of other natural products and plant‐derived phenolic acid monomers that may be obtained from the processing of lignin or lignocellulose waste streams (Table 1). Phenylpropenes (such as eugenol and isoeugenol) **5** **a**–**7** **a** and phenylpropanes **8** **a**–**9** **a** gave excellent conversions to *p*‐methylated products. Phenolic aldehydes **11** **a**–**15** **a** and phenolic acids **16** **a**–**22** **a** had variable conversions. In a few cases the WT enzyme performed better than the variants.

**Table 1:** Biocatalytic transmethylation of phenolic substrates mediated by EjOMT and variants.^[a]^

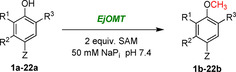						
						
Substrate	Z	R^1^	R^2^	R^3^	Best EjOMT variant	Conv. [%]^[b]^
**1** **a**, ferulic acid	CH=CHCOOH	OCH_3_	H	H	I133S/L138V/L342V	>99
**2** **a**, coumaric acid	CH=CHCOOH	H	H	H	I133S/L138V/L342V	>99
**3** **a**, sinapinic acid	CH=CHCOOH	OCH_3_	H	OCH_3_	I133S/L138V/L342V	97
**4** **a**, caffeic acid	CH=CHCOOH	OH	H	H	I133S/L138V/L342V	>99
**5** **a**, eugenol	CH_2_CH=CH_2_	OCH_3_	H	H	I133S/L138V/L342V	>99
**6** **a**, isoeugenol	CH=CHCH_3_	OCH_3_	H	H	I133S/L138V/L342V	>99
**7** **a**	CH_2_CH=CH_2_	OCH_3_	H	OCH_3_	I133S/L138V/L342V	96
**8** **a**	CH_2_CH_2_CH_3_	H	H	H	I133S	>99
**9** **a**	CH_2_CH_2_CH_3_	OCH_3_	H	H	I133S/L138V/L342V	>99
**10** **a**, vanillyl alcohol	CH_2_OH	OCH_3_	H	H	I133S	>99
**11** **a**, vanillin	CHO	OCH_3_	H	H	WT or I133S	>99
**12** **a**	CHO	H	H	H	WT or I133S	74
**13** **a**	CHO	OH	H	H	WT	43 (11b)
**14** **a**	CHO	H	OH	H	–	–^[c]^
**15** **a**, syringaldehyde	CHO	OCH_3_	H	OCH_3_	I133S/L138V/L342V	99
**16** **a**	COOH	H	H	H	I133S/L138V/L342V	99
**17** **a**	COOH	OH	H	H	I133S	99
**18** **a**, vanillic acid	COOH	OCH_3_	H	H	I133S or I133S/L138V	99
**19** **a**	COOH	H	OH	H	–	–^[c]^
**20** **a**, syringic acid	COOH	OCH_3_	H	OCH_3_	I133S/L138V/L342V	46
**21** **a**	CH_2_COOH	OH	H	H	WT	99
**22** **a**	CH_2_COOH	OCH_3_	H	OCH_3_	I133S/L138V/L342V	32

[a] Reaction conditions: 1 mM substrate, 2 mM SAM and 1 mg mL^−1^ purified EjOMT, 50 mM NaP_i_ buffer pH 7.4, 30 °C, 180 rpm, 18 h. [b] Determined by reverse‐phase HPLC. [c] No conversion.

**Scheme 2:**
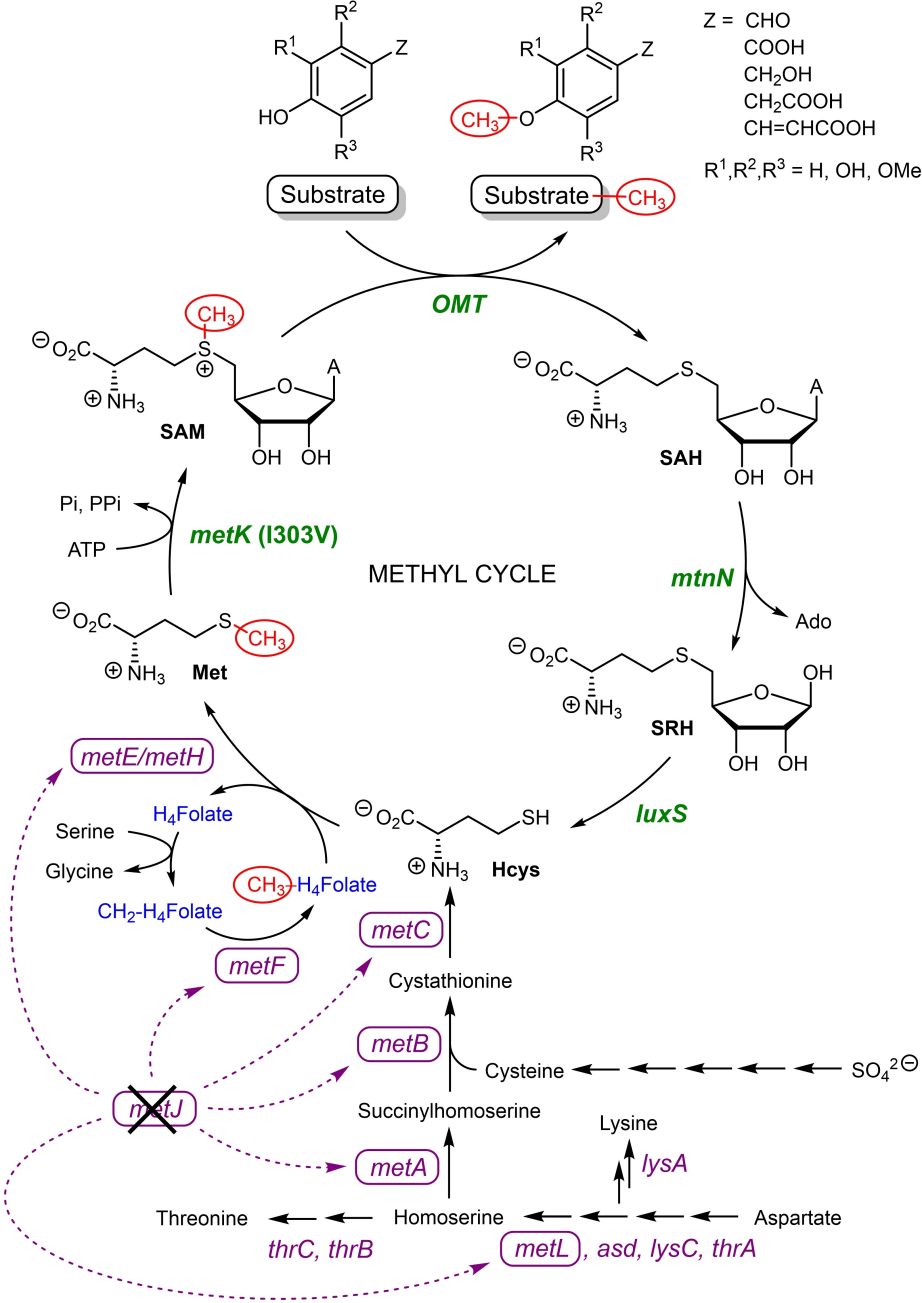
Optimised in vivo methylation reaction by engineering the methyl cycle. Overexpressed genes are indicated with green text. Negative feedback inhibition under the control of the methionine repressor protein MetJ (which has been disrupted) is indicated by purple dashed arrows, and the subsequent genes affected in purple boxes.

**Figure 1:**
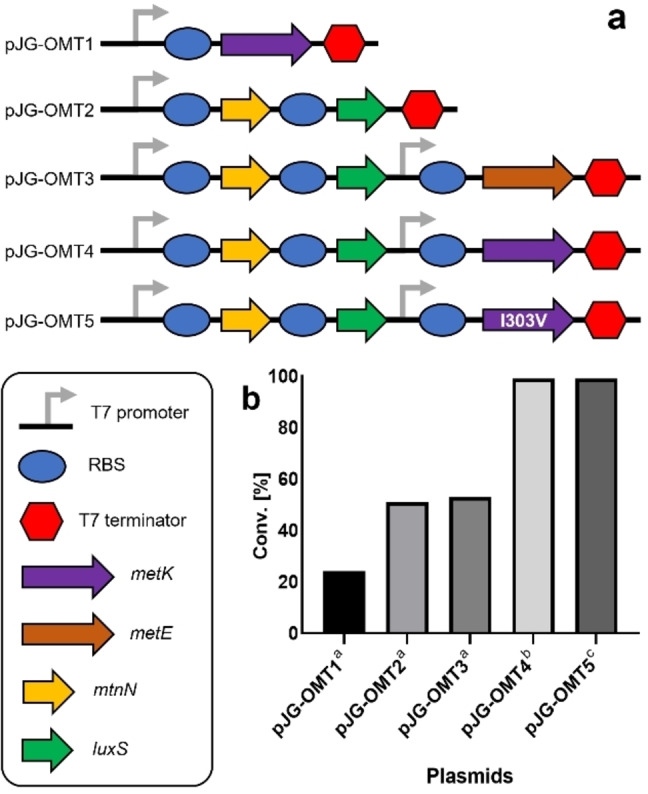
Metabolic engineering for ferulic acid methylation. a) Schematic representation of expressed genes in a medium‐copy plasmid. b) Percentage product conversion of ferulic acid using co‐expressed EjOMT I133S/L138V/L342V with accessory plasmids pJG‐OMT1 to 4 in BL21 (DE3). [a] 1 mM of **1** **a**, 10 equiv of l‐methionine, 3 % (w/v) wet cells, [b] 1 mM of **1** **a**, 10 equiv of dl‐methionine, 1.5 % w/v wet cells. [c] Δ*metJ* deficient BL21 (DE3) strain, 3 mM of **1** **a**, 1.6 equiv dl‐methionine, 0.7 % w/v wet cells.

SAM is a prohibitively expensive methyl group donor co‐substrate in the OMT‐catalysed methylation reaction. There have been several attempts at developing in vitro SAM regeneration systems that require the addition of multiple enzymes (making it difficult to optimise and resulting in low conversion yields^[31]^) as well as the use of chemical methylating agents (typically highly toxic methyl iodide^[32]^). An alternative approach was explored to tightly regulate the in vivo methyl cycle and circumvent several competitive feedback inhibitions for optimal methylation conditions (Scheme 2). We sought to identify these bottlenecks and overexpress in tandem the genes that are essential in the methyl cycle pathway (pJG‐OMT1 to 5, Figure 1), leading to an increase in product conversion.

Previous in vivo studies supplemented glucose and l‐methionine to the cell culture media^[33]^ and/or coexpressed the *E. coli* SAM synthase gene *metK* ^[34]^ to increase the methyl pool for higher conversions of methylated products, achieving some success, but with very low substrate loadings (<350 μM). Our initial studies with resting whole cells of EjOMT variant I133S/L138V/L342V (3 % w/v) in M9 media with 1 mM ferulic acid **1** **a** and 10 equiv of l‐methionine gave 24 % conversion to **1** **b**. Moreover, coexpression with an *E. coli* SAM synthase *metK* in a medium‐copy accessory plasmid (pJG‐OMT1) gave comparable results (Figure 1b).

Low conversions can be attributed to the concomitant formation of co‐product *S*‐adenosyl‐l‐homocysteine (SAH), a known product inhibitor which obstructs reversibly the active site of methyltransferases.^[35]^ To expedite continuous SAH removal, two endogenous *E. coli* genes downstream from SAH synthesis (*S*‐adenosylhomocysteine nucleosidase *mtnN* and *S*‐ribosylhomocysteine lyase *luxS*) were assembled and homologously expressed in a synthetic operon with strong ribosome binding sites (pJG‐OMT2). A two‐fold increase in product conversion was observed (51 %) within 24 h, and an additional 18 h gave full conversion to the methylated product. The degradation of SAH was deemed essential to the reaction cycle by increasing metabolic flux to homocysteine,a recycled precursor for methionine synthesis, while adenosine is salvaged in the adenylate pool.

**Figure 2:**
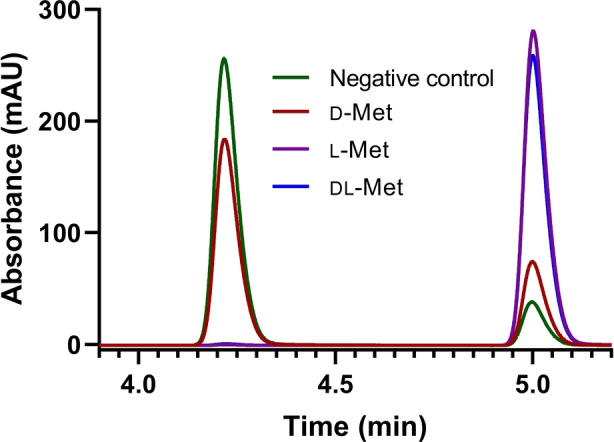
HPLC analysis of the one‐pot four‐enzyme cascade reactions using cell lysates of coproduced proteins MetK I303V, MtnN, LuxS (pJG‐OMT5) and purified EjOMT I133S/L138V/L342V. The reaction was performed for 18 h, with 1.5 equiv of ATP and methionine and 1 mM ferulic acid substrate **1** **a** (4.2 min), to yield methylated product **1** **b** (5.0 min).

Inspired by these results, we investigated gene products for the enhanced synthesis of SAM from homocysteine: cobalamine‐independent methionine synthase *metE* from *Catharanthus roseus* ^[36]^ (which has higher specific activities than the *E. coli* homolog) and *E. coli* SAM synthase *metK*. These genes were individually subcloned into plasmid pJG‐OMT2 containing the SAH degrading genes but under the control of a separate T7 promoter, and product conversions were assessed with the coexpression of the EjOMT mutant I133S/L138V/L342V. Unfortunately, the inclusion of a methionine synthase *metE* (pJG‐OMT3) gave comparable conversions to the accessory genes involved in SAH degradation alone (pJG‐OMT2). Most surprising was the addition of the *E. coli* SAM synthase *metK* gene containing plasmid (pJG‐OMT4) which gave full conversion to **1** **b** in 18 h. We further attempted to improve substrate loading, reduce resting cell biomass and lower the concentration of supplemented methionine, for an efficient whole‐cell platform. To overcome the current limitations we firstly addressed the product inhibition of *E. coli* SAM synthase MetK,[^37a^, ^37b^] which may have an overall effect on metabolic flux to SAM synthesis and subsequent incomplete transmethylations. Previous studies had engineered a SAM synthase MetK from *Bacillus subtilis* with reduced product inhibition,^[38]^ the active site amino acid residue I317 was identified to interact with the methyl group of SAM and possibly hindering the correct orientation of the substrate bound in the active site. Substituting the I317 residue with valine showed a remarkably enhanced activity with both l‐ and dl‐methionine and was not inhibited by the d‐enantiomer. Another research group mutated the same conserved isoleucine amino acid found in *E. coli* MetK (I303) to valine and observed a 4‐fold increase in activity with decreased product inhibition.^[39]^ We introduced into the plasmid‐borne construct SAM synthase *metK* mutant I303V (pJG‐OMT5), and performed an in vitro enzyme cascade using cell lysates of coproduced proteins (MetK I303V, MtnN and LuxS) in the presence of ATP and methionine to produce SAM in situ, as well as purified EjOMT (I133S/L138V/L342V) variant and ferulic acid substrate **1** **a**. This enzyme cascade gave full conversion with l‐ and dl‐methionine (>99 %) but, most surprisingly, even d‐methionine produced twice as much methylated product (20 %) than the negative control with methionine omitted (Figure 2).

Our one‐pot four‐enzyme cascade revealed that the engineered methyltransferase variant exhibited low methylation activity with the biologically inactive (*R*,*S*)‐SAM stereoisomer^[40]^ generated by the MetK I303V variant with d‐methionine. We applied our in vitro cascade as a whole cell system reducing wet cell loading to 1.5 % w/v, using 10 equiv of inexpensive dl‐methionine and incubating for 24 h at 30 °C, affording >99 % conversion to **1** **b**. Increasing substrate loading to 2 mM ferulic acid or lowering dl‐methionine to 5 mM in the reaction media gave 49 % conversion to **1** **b** after 48 h. Interestingly, the decarboxylation byproduct 4‐vinylguaiacol was also detected (6 %), possibly arising from metabolic bottlenecks, such as the methyltransferase EjOMT reaching suboptimum turnover rates due to restricted SAM availability. Presumably, the endogenous flavin prenyltransferase UbiX/UbiD system,[^41^, ^42^] known to decarboxylate ferulic acid, can compensate for the metabolic burden from ferulic acid toxicity by converting it to 4‐vinylguaiacol.

In order to circumvent controlled methionine and SAM availability in the cell we focused on the methionine transcriptional repressor protein MetJ, which regulates the mRNA production of the genes responsible for the intracellular methionine with the presence of its corepressor SAM.[^43a^, ^43b^] Negative regulation was relieved by deleting the *metJ* gene that derepresses the methionine regulon leading to an increase in l‐methionine availability for SAM production (Scheme 2).[^44a^, ^44b^]

Our newly improved strain Δ*metJ* BL21(DE3) containing the EjOMT mutant I133S/L138V/L342V and pJG‐OMT5 plasmid performed at high substrate loadings (3 mM, **1** **a**) with 1.6 equiv of dl‐methionine supplement (5 mM) and reduced *E. coli* resting cell biomass 0.7 % w/v gave >99 % conversion to **1** **b** at a titre of 0.6 g L^−1^ in 48 h.

**Scheme 3:**
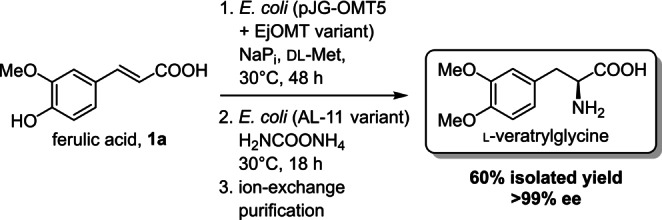
Preparative scale synthesis of l‐veratrylglycine from ferulic acid.

Lastly, to demonstrate the synthetic application of these enzyme we performed a preparative scale biotransformation to produce l‐veratrylglycine in a one‐pot, two‐step whole cell telescopic cascade (Scheme 3). In the first step the methylation of ferulic acid to **1** **b** was monitored until complete conversion (48 h). Subsequently, additional *E. coli* resting cells (0.8 % w/v) overproducing the ammonia lyase AL‐11 variant Q84V and solid ammonium carbamate (to reach a final concentration of 4 M) were added to the suspension. The AL‐11 Q84V variant has been previously identified as the best candidate for the hydroamination of **2** **b** in a previous screening.^[28]^ The mixture was incubated at 30 °C, until HPLC analysis of the reaction media showed complete conversion of **2** **b** to l‐veratrylglycine (18 h). The amino acid product was readily purified by adsorption on ion‐exchange resin, resulting in a 60 % (189 mg) isolated yield, confirmed by ^1^H NMR. The product was obtained in excellent enantiopurity (>99 % ee). Interestingly, we did not observe accumulation of intracellular methylated intermediate **2** **b** or l‐veratrylglycine: presumably, the AaeAB efflux pump^[45]^ in *E. coli* that is known to transport hydroxylated aromatic carboxylic acids can effectively remove the toxic intermediate metabolite **2** **b** from the cell. Moreover, exposure of resting *E. coli* cells to harsh chemical reaction conditions (4 M ammonium carbamate) may permeabilise the cell membrane enhancing transport of metabolites for enzyme catalysis in the production of l‐veratrylglycine.

## Conclusion

In summary, we have successfully engineered a putative 4‐*O*‐methyltransferase variant to accept a variety of phenolic substrates which can be derived from lignin. Furthermore, we developed a SAM cofactor regeneration system focused on overexpressing genes in the methyl cycle pathway with the supplementation of only 1.6–2.0 equiv of exogenous dl‐methionine. We anticipate this new strain can be used for other methylation pathways using *E. coli* as the microbial host for highly sought after methylated products. We have also demonstrated for the first time a preparative‐scale multistep cascade for the production of the l‐DOPA precursor l‐veratrylglycine from lignin‐derived ferulic acid in high yield and purity. Further work is currently underway to develop a fully integrated enzymatic process to demethylate l‐veratrylglycine to l‐DOPA using cytochrome P450 aromatic O‐demethylase^[46]^ for a greener, more effective, and sustainable process.

## Conflict of interest

The authors declare no conflict of interest.

## Supporting information

As a service to our authors and readers, this journal provides supporting information supplied by the authors. Such materials are peer reviewed and may be re‐organized for online delivery, but are not copy‐edited or typeset. Technical support issues arising from supporting information (other than missing files) should be addressed to the authors.

Supporting Information
