# Supplementary material for: One‐Pot Biocatalytic In Vivo Methylation‐Hydroamination of Bioderived Lignin Monomers to Generate a Key Precursor to L‐DOPA
Source: Angew Chem Weinheim Bergstr Ger. 2022 Jan 10;134(8):e202112855. doi: 10.1002/ange.202112855 (PMC10947412; doi:10.1002/ange.202112855)
Supplement: Supplementary file 1 — Supporting Information [file ANGE-134-0-s001.pdf]

Angewandte Chemie
Eine Zeitschrift der Gesellschaft Deutscher Chemiker

Supporting Information

One-Pot Biocatalytic In Vivo Methylation-Hydroamination of Bioderived Lignin Monomers to Generate a Key Precursor to L-DOPA

*J. L. Galman, F. Parmeggiani, L. Seibt, W. R. Birmingham, N. J. Turner**

General methods

All commercially available reagents, analytical standards and solvents were purchased from Merck KGaA (Darmstadt, Germany), Alfa Aesar (Morecambe, England), VWR (Lutterworth, England) or Fluorochem Ltd (Hadfield, UK) and used without further purification. *Escherichia coli* DH5α and BL21 (DE3) cells, NEBuilder® HiFi DNA Assembly Master Mix, Q5® High-Fidelity 2X Master Mix, 1 kb Plus DNA Ladder and all restriction enzymes were purchased from New England Biolabs (Ipswich, MA, USA). Oligonucleotides were synthesized at Eurofins, genomics (Ebersberg, Germany). Plasmid DNA Miniprep kit, DNA gel extraction kit and PCR purification kits were purchased from Qiagen (Düsseldorf, Germany). HPLC filter vials 0.45uM PVDF with a pre-slit cap were bought from Thomson (California, USA). Expression vector pET-28b was purchased from Novagen (Darmstadt, Germany) and was used for gene expression. Plasmids pKD46 and pKD3 was obtained from the E. coli Genetic Resources at Yale CGSC. Genes *EjOMT* and *Cr-metE* were codon optimized and purchased from Life Technologies Limited (Paisley, UK) as GeneArt Strings™ DNA fragments. Plasmid DNA was transformed into *E. coli* cells via heat shock method or electroporated using a Gene Pulser Xcell Microbial System (Bio-Rad) in 0.1 cm cuvettes and set to 1.80 kV. Plasmids constructs and PCR amplicons were sequence verified at Eurofins MWG Operon (Ebersberg, Germany). Plasmid maps were constructed and visualised using Snapgene (San Diego, USA).

PCR thermocycling conditions

PCR amplifications were performed using Q5® High-Fidelity 2X Master Mix protocol[1]. All primers were designed to have annealing temperature of 60°C. The following thermocycling conditions were used: (1) 98°C for 1 min, (2) 25 cycles: 98°C for 30 s, 60°C for 30 s, and 72°C 20-30 seconds/kb (adjusted accordingly), and (3) 72°C for 5 min. The amplified DNA was purified either using an agarose gel or a Qiagen PCR purification kit.

Codon optimized EjOMT DNA sequence

The protein sequence ID for *Eriobotrya japonica O*-methyltransferase is UNIPROT: A0A1B4Z3W1 or GenBank: BAV54107.1. The lower-case sequence represents the DNA base overhangs that anneal to the DNA fragment from the cut plasmid vector pET28b using NdeI/XhoI restriction enzymes. The two constructs were assembled using NEBuilder® HiFi DNA Assembly Master Mix[2] following the manufacturer's protocol and subsequently transformed into commercial DH5α *E.coli* cells and plated onto kanamycin plates.

```
gccgcgcggcagccatATGGGGCTTAGCAACGGAGACGAAGTCGGCGCGACTTCACATGAACTTCTTGGTGCTCA
GGCGCAGATTTGGAATCATATGTTCCAGTTCATTAATAGTATGGCGTTAAAGTGTGCGGTTCAGTTGGGAATCCC
CGACGTTATTCATAATCACGGGCAGCCCATCAGTTTGTCCGAACTTATCGCTGCTCTTAATGTCCATCCATCGAA
GGCGCATTTTGTTTCCCGCTTAATGCGTATTCTTGTTCACTCAGATTTCTTTGCTCAGCACCACCACGTCCATCA
TGATTGTGACGATGTTGAGGAGGAAGAAGCTGTCGTCCTTTACTCGCTGACACCGACCAGCCGTCTGTTATTGAA
AGATGGCCCATCTAATACGACTCCGCTTGTATTGATGATCTTGGACCCGGTGTTGACTACTCCGTTCCATCTTAT
GGGCGCATGGCTTCAAATGAATGGCGGGGATGATCCGGCCACGATTTGTACGCCCTTTGAGATGGAAAATGGTAT
GCCATTCTGGGACCTGGCAGCGCAAGAACCACGTTTTGGCAACTTGTTCGACGAGGCCATGGAAGCAGATTCGAA
GTTGTTGGGCCGCGAGGTAGTGGAAGAATGCGGAGGGGTTTTCGAGGGTCTGAAATCTCTTGTAGATGTAGGTGG
AGGGACTGGCACCATGGCTAAAGCCATTGCCAATGCTTTCCCATCCATTAATTGCATCGTATTCGATCAACCACA
TGTAGTTGCCGATCTTCAAGGGACTACGCACAACCTTGGGTTTGTTGGCGGAGATATGTTCGTTGAAATCCCCCC
TGCGAATGCGATTTTGTTGAAGTGGATTCTTCACGATTGGTCGGACGAGGAATCGGTCAAGATTCTGAAGAACTG
TCGCGAAGCCATTTTGTTGTCTAAAAATAATGAGGGGAACAAGAAAATCATCATCATTGACATTGTCGTAGGGCA
CGTGGATAATAAAGAAAAGATGGTAGACAAAAAGTCGATTGAAACCCAATTGATGTTCGACATGCTTATGATGTC
CACAGTTACTGGTAAGGAGCGCTCAGAAAGTGAATGGAAAAAGATCTTCCTGGCAGCCGGTTTTACCCATTATAA
CATCACTCATGCTTTCGGTTTCCGTTCACTGATCGAATTATACCCAAGTAAGTGActcgagcaccaccaccacca
```

Construction of plasmids pJG-OMT1-5

Plasmids pJG-OMT1-5 were constructed from the backbone of the commercial pACYC-Duet plasmid with the chloramphenicol resistance marker substituted to an ampicillin resistance marker to yield pJG-OMT.

1. pJG-OMT1

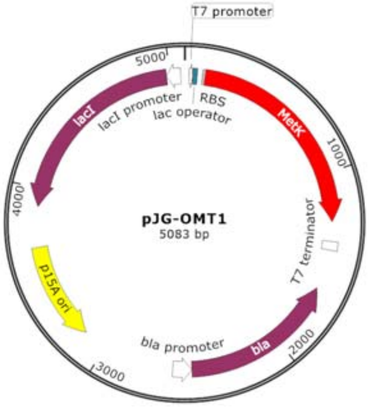

Plasmid pJG-OMT was cut using restriction enzymes NdeI/XhoI. The *metK* gene was cloned from an *E. coli* K-12 strain using the following primers (the lower-case sequence represents the DNA base overhangs):

MetK Fw: ttaagtataagaaggagatatacatATGGCAAAACACCTTTTTACGTCCG
MetK Rv: gcggtttctttaccagactcgagTTACTTCAGACCGGCAGCATCG

The cloned *metK* gene and cut pJG-OMT vector was assembled following the NEBuilder® HiFi DNA Assembly Master Mix protocol[2]. Finally, multiple cloning site 1 (MCS1) was deleted via the inverse PCR[3] method using the following primers:

ΔMCS1 Rv: AGGGAGAGCGTCGAGATCC
ΔMCS1 Fw: TTGTACACGGCCGCATAATC

2. pJG-OMT2

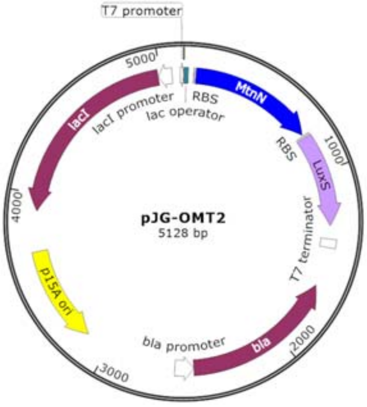

The *mtnN* gene (genbank: U24438.1) and *luxS* gene (genbank: AAC75734.1) was cloned from an *E. coli* K-12 strain using the following primers (RBS underlined):

MtnN Fw: GAGATATACCATGAAAATCGGCATCATTGGTGCA
MthN Rv: TAACAACGGCATTATATATCTCCTTAAGCTTTTAGCCATGTGCAAGT
LuxS Fw: GGAGATATATAATGCCGTTGTTAGATAGCTTCACAG
LuxS Rv: GCGGCCGCCTAGATGTGCAGTTCCTGCAACTTC

Plasmid pJG-OMT was amplified using the following primers:

Vector Fw: CATCTAGGCGGCCGCATAATGCTTAAG
Vector Rw: CCGATTTTCATGGTATATCTCCTTATTAAAGTTAAACAAAATTATTTCTACAGGGG

The amplicons were assembled following the NEBuilder® HiFi DNA Assembly Master Mix protocol.[2] Finally, multiple cloning site 2 (MCS2) was deleted via the inverse PCR[3] method using the following primers:

ΔMCS2 Rv: CCGCTGAGCAATAACTAGC
ΔMCS2 Fw: GATTATGCGGCCGTGTACAA

3. pJG-OMT3

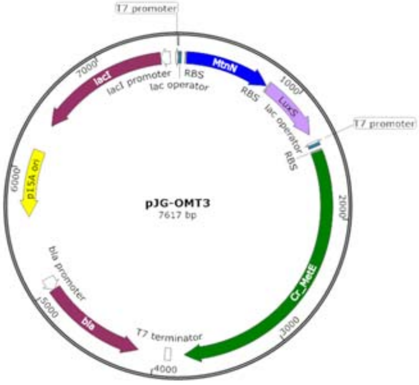

Codon optimized Cr-MetE DNA sequence (protein sequence UNIPROT ID: Q42699)

```
gttaagtataagaaggagatatacatATGGCCAGCCATATTGTTGGTTATCCGCGTATGGGTCCGAAACGTGAAC
TGAAATTTGCACTGGAAAGCTTTTGGGACAAAAAAAGCAGCGCAGAAGATCTGCAGAAAGTTGCAGCCGATCTGC
GTAGCAGCATTTGGAAACAAATGGCAGATGCAGGCATTAAATACATTCCGAGCAATACCTTCAGCTATTATGATC
AGGTTCTGGATACCGCAACCATGCTGGGTGCAGTTCCGCCTCGTTATAACTTTGCCGGTGGTGAAATTGGTTTCG
ATACCTATTTTAGCATGGCACGTGGTAATGCAAGCGTTCCGGCAATGGAAATGACCAAATGGTTTGATACCAACT
ACCATTATATTGTGCCGGAACTGGGTCCGGAAGTGAATTTTTCTTATGCAAGCCATAAAGCCGTGAACGAATATA
AAGAAGCAAAAGAACTGGGCGTTGATACCGTTCCGGTTCTGGTTGGTCCGGTTACCTTTCTGCTGCTGAGCAAAC
CGGCAAAAGGTGTTGAAAAAACATTTCCGCTGCTGTCACTGCTGGATAAAATTCTGCCGGTTTATAAAGAGGTTA
TCGGCGAACTGAAAGCAGCCGGTGCAAGCTGGATTCAGTTTGATGAACCGACCCTGGTGCTGGATCTGGAAAGCC
ATCAGCTGGAAGCATTTACCAAAGCATATAGCGAACTGGAAAGTACCCTGAGCGGTCTGAATGTTATTGTGGAAA
CCTATTTTGCAGACATTCCGGCAGAAACCTATAAAATCCTGACCGCACTGAAAGGTGTTACCGGTTTTGGTTTTG
ATCTGGTTCGTGGTGCAAAAACCCTGGATCTGATTAAAGGTGGTTTTCCGAGCGGTAAATACCTGTTTGCGGGTG
TTGTTGATGGTCGTAATATTTGGGCAAATGATCTGGCAGCAAGCCTGAGCACCCTGCAGAGCCTGGAAGGCATTG
TTGGTAAAGATAAACTGGTTGTTAGCACCAGCTGTAGCCTGCTGCATACCGCAGTCGACCTGGTTAATGAACCGA
```

AACTGGATAAAGAGATTAAAAGCTGGCTGGCATTTGCAGCACAGAAAGTGGTTGAAGTTAATGCACTGGCAAAAG
CACTGGCAGGCGAAAAAGATGAAGCATTTTTTAGCGAAAATGCAGCAGCACAGGCAAGCCGTAAAAGCAGTCCGC
GTGTTACCAATCAGGCAGTTCAGAAAGCAGCAGCAGCCCTGCGTGGTAGCGATCATCGTCGTGCAACCACCGTTA
GCGCACGTCTGGATGCACAGCAGAAAAAACTGAACCTGCCGGTTCTGCCGACCACCACCATTGGTAGCTTTCCGC
AGACCCTGGAACTGCGTCGTGTTCGTCGTGAGTATAAAGCCAAAAAAATCTCCGAGGATGATTATGTGAAAGCGA
TCAAAGAAGAAATCAGCAAAGTCGTGAAACTGCAAGAGGAACTGGATATTGATGTTCTGGTGCATGGTGAACCTG
AACGTAATGATATGGTGGAATATTTTGGTGAACAGCTGAGCGGTTTTGCATTTACCGCAAATGGTTGGGTTCAGA
GCTATGGTAGCCGTTGTGTTAAACCGCCTATTATCTATGGTGATGTTAGCCGTCCGAATCCGATGACCGTTTTTT
GGAGCCAGACCGCACAGAGCATGACCAAACGTCCGATGAAAGGTATGCTGACCGGTCCGGTGACAATTCTGAATT
GGAGCTTTGTTCGTAATGATCAGCCTCGTTTTGAAACCTGTTATCAGATTGCACTGGCCATTAAAGATGAGGTTG
AGGACCTGGAAAAAGCCGGTATTAATGTGATTCAGATCGATGAAGCCGCACTGCGTGAAGGTCTGCCGCTGCGTA
AAGCAGAACATGCATTTTATCTGGATTGGGCAGTTCATAGCTTTCGCATTACCAATCTGCCTCTGCAGGATACCA
CCCAGATTCATACCCACATGTGTTATAGCAACTTCAACGATATCATCCACAGCATCATTGATATGGATGCAGATG
TGATGACCATTGAAAATAGCCGTAGCAGCGAAAAACTGCTGAGCGTGTTTCGTGAAGGTGTTAAATATGGTGCAG
GTATTGGTCCGGGTGTGTATGATATTCATAGTCCGCGTATTCCGAGTACCGAAGAAATTGCAGATCGCATTAACA
AAATGCTGGCCGTGCTGGATACCAATATTCTGTGGGTTAATCCTGATTGTGGTCTGAAAACCCGTAAATATGCAG
AAGTGAAACCTGCCCTGGAAAATATGGTTAGCGCAGCAAAACTGATTCGTACCCAGCTGGCAAGCGCAAAACTCG
AGTAAcctcgagtctggtaaagaaaccgc

The lower-case sequence represents the DNA base overhangs that anneal to the DNA fragment from the cut plasmid pJG-OMT2 using NdeI/XhoI restriction enzymes. The codon optimised *Cr-metE* gene and cut pJG-OMT2 vector was assembled following the NEBuilder® HiFi DNA Assembly Master Mix protocol[2].

4. pJG-OMT4

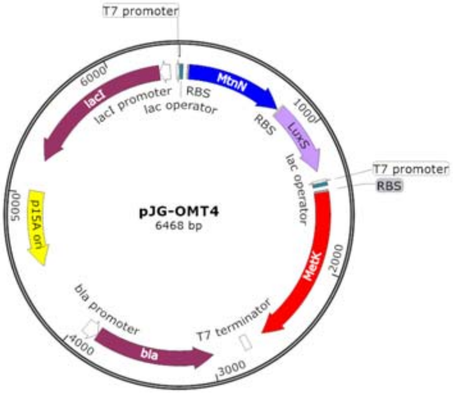

The second multiple cloning site (MCS2) of the pJG-OMT2 vector was cut using NdeI/XhoI restriction enzymes. The *Ec metK* gene was subcloned into pJG-OMT2 using the same protocol in the construction of pJG-OMT1 (See above).

5. pJG-OMT5

Point mutation (I303V) was introduced in the *Ec-metK* gene using the quikchange method[4] and the following primers with mutation in bold:

MetK I303V Fw: GGTTTCCTACGCA**GTA**GGCGTGGCTGAACC
MetK I303V Rv: GGTTCAGCCACGCC**TAC**TGCGTAGGAAACC

Afterward, 10 U of DpnI was added to the reaction mixture and was incubated for 1 h at 37 °C to digest parental DNA and 2uL used to transform *E. coli* DH5α cells.

Deletion of *metJ* gene in *E. coli* BL21 (DE3) cells:

The disruption of the methionine repressor protein MetJ was based on the λ red homologous recombination procedure for creating a knock-out mutant as described by Datsenko *et al*.[5], with several modifications. A DNA fragment containing a selectable antibiotic resistance gene: chloramphenicol was amplified by PCR using plasmid pKD3 as a template and the primers listed below which contained 50 homologous base pair extensions. Arabinose induced BL21 (*DE3*) cells containing the λ red recombinase expression plasmid pKD46, was electroporated with 100 ng of amplified DNA (pretreated with DpnI) left to incubate in an orbital shaker at 37°C for 2 hr and subsequently plated on LB agar in the presence of 30 μg mL^{-1} of chloramphenicol. Colony PCR was performed to confirm the incorporation of the chloramphenicol resistance gene in *E. coli* BL21 (DE3) genome.

Primers for amplification chloramphenicol resistance cassette from pKD3.

MetJ Fw:
GCTGTGCGAAGCGTTTCTGCATGCCTTTACCGGGCAACCTTTGCCGGATGGTGTAGGCTGGAGCTGCTTC
MetJ Rv:
CTCGTTGTTTATGCCGGATGCGGCGTGAACGCCTTATCCGGCCTACAAGTATGGGAATTAGCCATGGTCC

Primers for Colony PCR to confirm *metJ* gene disruption.

Metj Seq Fw: GCGCGGTTCATTAAATCCGG
CAM Seq Rv: GGCGAAAATGAGACGTTGATCG

Protein expression and purification

Plasmid pET28b-EjOMT was transformed into *E. coli* BL21 (*DE3*). A fresh colony was used to inoculate LB medium (3 mL) containing kanamycin (50 µg mL^{-1}). This freshly prepared overnight culture was grown at 200 rpm at 37°C, and was used to inoculate 500 mL of LB medium supplemented with kanamycin (50 µg mL^{-1}) in a 2 L baffled flask at 200 rpm at 37°C. The recombinant protein expression was induced by adding isopropyl-β-D-1-thiogalactopyranoside (IPTG) (0.5 mM, final) when OD_{600} reached 0.8-1.0. The cell cultures were then incubated at 18°C for 18 h. The cells were harvested by centrifugation at 4°C (3,250 g, 20 min) and were resuspended (1g in 10 mL) in lysis buffer (50 mM Tris-HCl, 5 mM imidazole, pH 7.0) and lysed in an iced bath by ultra-sonication by Soniprep 150 (20 s on, 20 s off, for 20 cycles, at 30% amplitude). After centrifugation (4°C, 16,000 g, 20 min) the clarified lysate was used for protein purification via a Ni-NTA agarose column. The bound enzyme was washed with 20 mL wash buffer (50 mM Tris-HCl, 30 mM imidazole pH 8.0), and eluted with 50 mM Tris-HCl, 250 mM imidazole at pH 7.0. The collected fractions were concentrated in Vivaspin™ filter spin membrane columns (10,000 MWCO). The purified enzyme was washed several times and buffered exchanged with 50 mM potassium phosphate buffer at pH 7.4. The purity was analysed by SDS/PAGE and the protein was more than 95% pure and the protein stock was determined by the Bradford assay using bovine serum albumin as standard.

Homology model of EjOMT

YASARA (version 18.4.24) was used for energy minimization. Overlay of the lowest energy homology model and LnCa9OMT structure was performed and visualized with PyMol Molecular Graphics System, Schrçdinger,LLC.

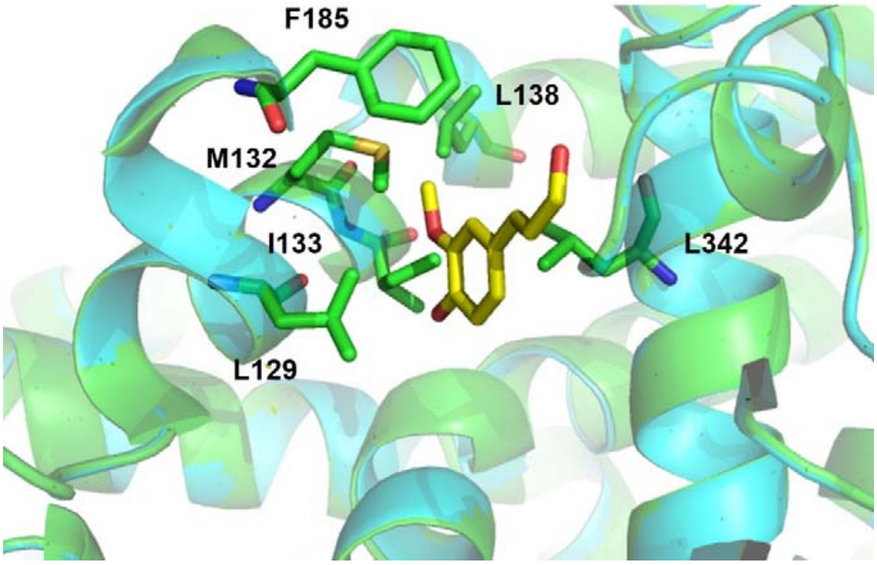

LnCa9OMT from *Linum nodiflorum* (pdb: 4E70) shares a 42% protein sequence identity with EjOMT. Overlay of active sites of bound coniferol ligand (yellow) of LnCa9OMT (cyan) and EjOMT (green) in stereo view. Six amino acids were selected (L129, M132, I133, L138, F185, L342) for alanine-valine scanning. Hydrogen bonding of amino acid residue S122 in LnCa9OMT was restored in EjOMT mutating the equivalent position I133 to serine.

Analytical scale biotransformations and LC-MS analysis

Unless otherwise specified, all assays were performed in 2 mL Eppendorf tubes, at 30°C in biotransformation buffer 50 mM KPi at pH 7.4. To the addition of 1mM substrate 1a-23a (from a 50 mM DMSO stock solution) was added 2 mM S-Adenosyl-L-methionine disulfate tosylate (from a 50 mM stock solution) and purified EjOMT (1 mg mL^{-1}, purified as described above) in a final volume of 0.5 mL. After 18 h, the reaction mixture was quenched by adding 0.5 mL of MeOH and centrifuged at 13K rpm for 5 mins to pellet protein debris. Finally, 400 µL of supernatant was passed through a Thomson HPLC filter vial (0.45uM PVDF).

Conversions were calculated by HPLC analysis on an Agilent 1260 Series system equipped with a quaternary pump VL (DEAEX00490), Vial sampler (G7129A), multicolumn thermostat (G7116A), a Waters® SPHERISORB® 5 µm ODS2 4.6 x 250 mm analytical column and a diode UV array detector (G7115A). Separation conditions: 1 mL min^{-1} flowrate, mobile phases: H_2O + 0.1% v/v TFA, MeCN + 0.1% v/v TFA. Gradient: Starting at 80:20 (H_2O:MeOH) then 20:80 over 6 min, then 80:20 over 1 min then hold for 4 min. Temperature: 35 °C. Detection wavelength: 210 nm, 254 nm and 324 nm. Injection volume: 5 µL. Products were confirmed via chemical standards.

Whole cell biocatalysis

E.coli strains were made electrocompetent according to published protocols[6]. 100uL of electrocompetent cells containing 1 µL (60 ng μL^{-1}) of EjOMT mutant I133S/L138V/L342V was co-transformed with 1 µL (60 ng μL^{-1}) of pJG-OMT5 via electroporation. The cells were treated to 1 mL of SOC media and incubated in an orbital shaker for 1 hr at 37° C and plated on appropriate antibiotic LB plates. For example, BL21 (DE3) Δ*metJ*::cam cells was electroporated with plasmids EjOMT mutant I133S/L138V/L342V and pJG-OMT5 and plated onto agar plates containing: 25 µg mL^{-1} carbenicillin 15 µg mL^{-1} kanamycin and 15 µg mL^{-1} chloramphenicol. 3 mL of LB media was inoculated with a single colony and left to grow overnight at 37 °C. The seed cultures were then used to inoculate 500 mL of LB media in 2L baffled flasks with appropriate antibiotics and cultivated at 37 °C in an orbital shaker (180 rpm). Once the OD_{600} reached 0.8, the cells were induced by 0.5 mM IPTG and the temperature was reduced to 18 °C and left for 18 hrs. The cell culture was harvested by pelleting at 4000 rpm and washed twice with fresh 25 mL LB media. The resting *E. coli* cells (3.6g) were resuspended in M9 media (500 mL) and placed in a 2 L Erlenmeyer flask containing 0.5 mM IPTG with appropriate antibiotics for plasmid maintenance. DL methionine powder was added directly to flask at final concentration of 10 mM and 1.25 mL of ferulic acid substrate (5mM final concentration) in DMSO from a 2M stock solution was added and placed in at orbital shaker at 30 °C (180 rpm). The whole cell biotransformation was monitored and sampled periodically by removing 0.5 mL of cell culture and quenching the reaction with 0.5 mL of methanol and centrifuged at 13K rpm to remove cell debris. The supernatant was passed through a Thomson filter vial (0.45uM PVDF) and analyzed via HPLC.

Representative HPLC traces

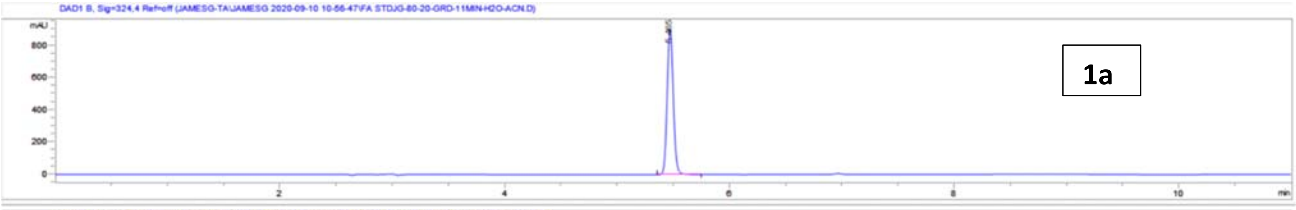

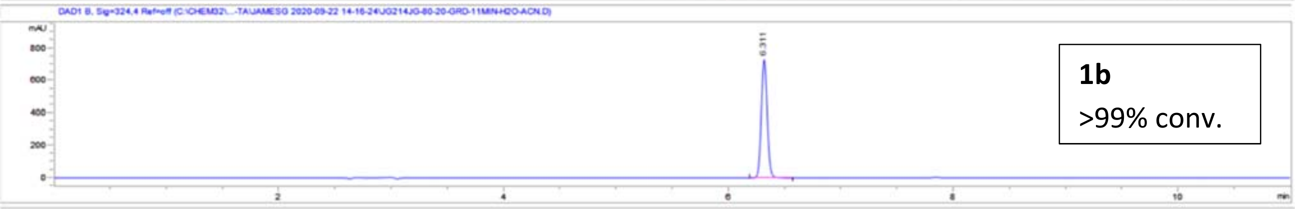

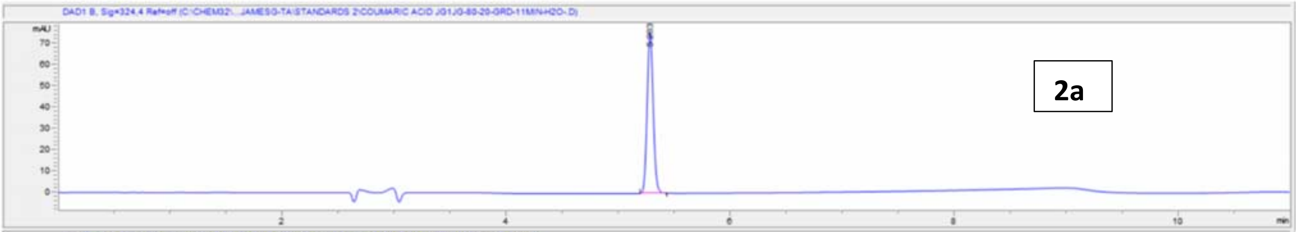

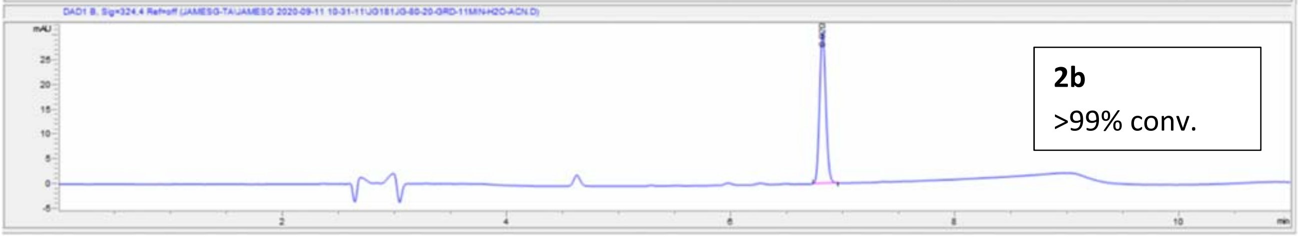

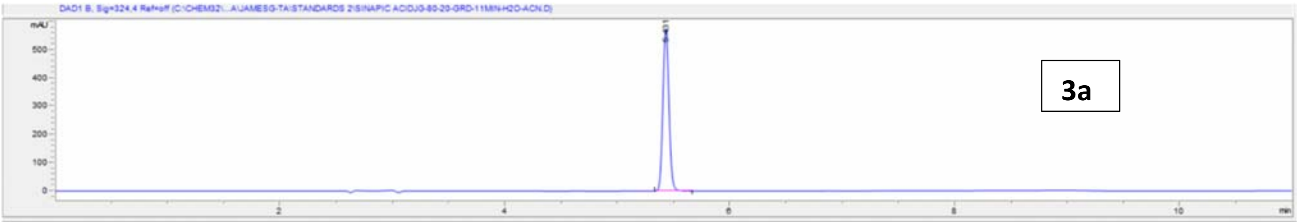

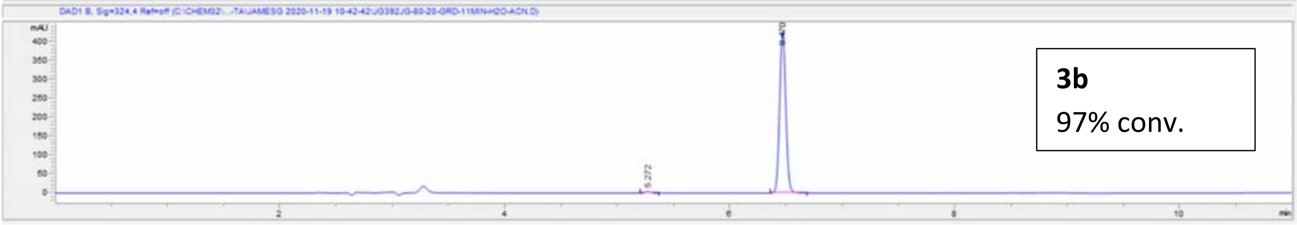

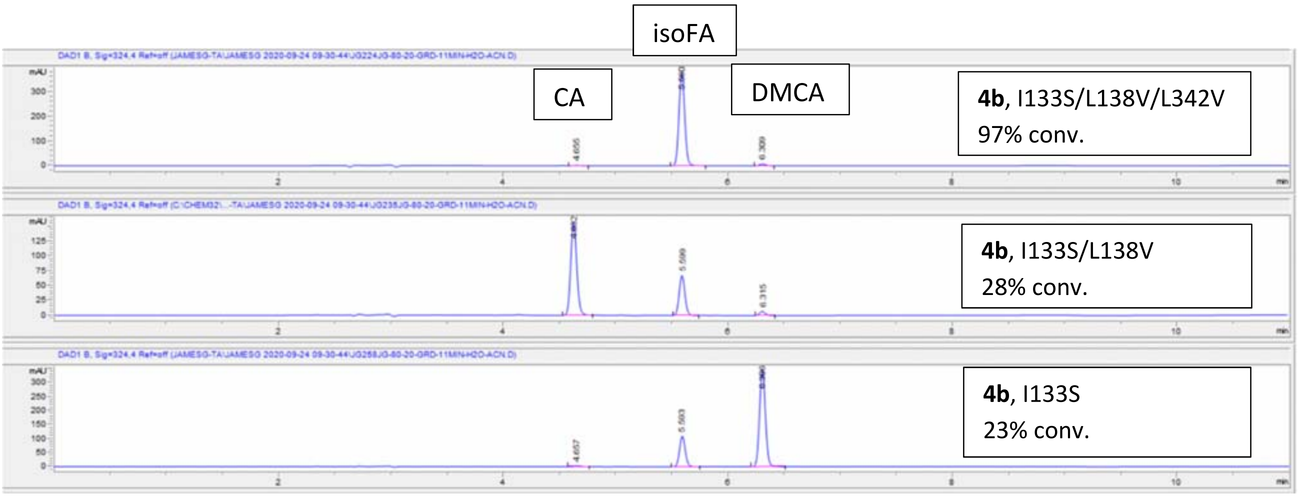
isoFA
CA
DMCA
4b, I133S/L138V/L342V
97% conv.
4b, I133S/L138V
28% conv.
4b, I133S
23% conv.

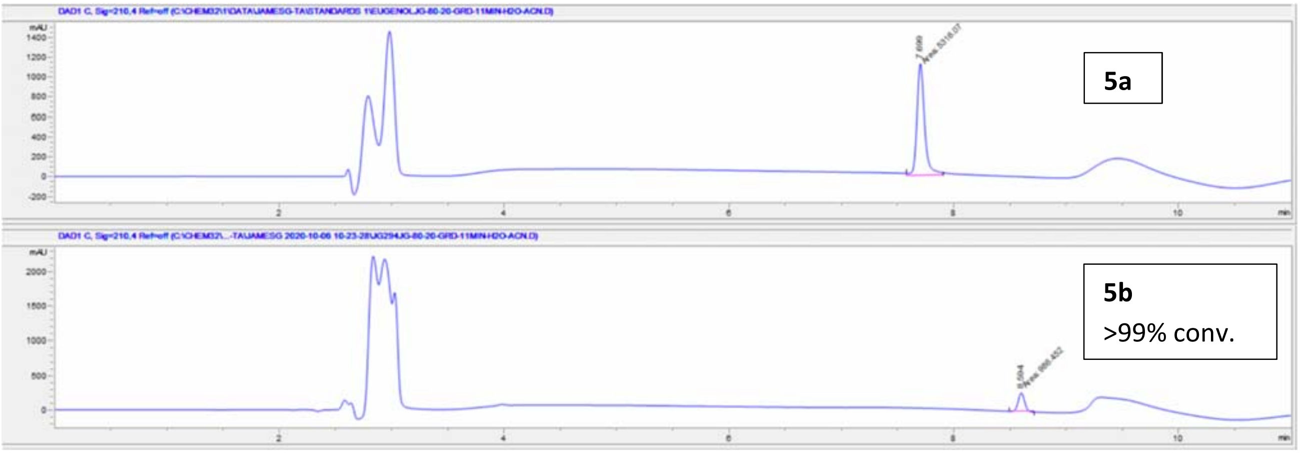
5a
5b
>99% conv.

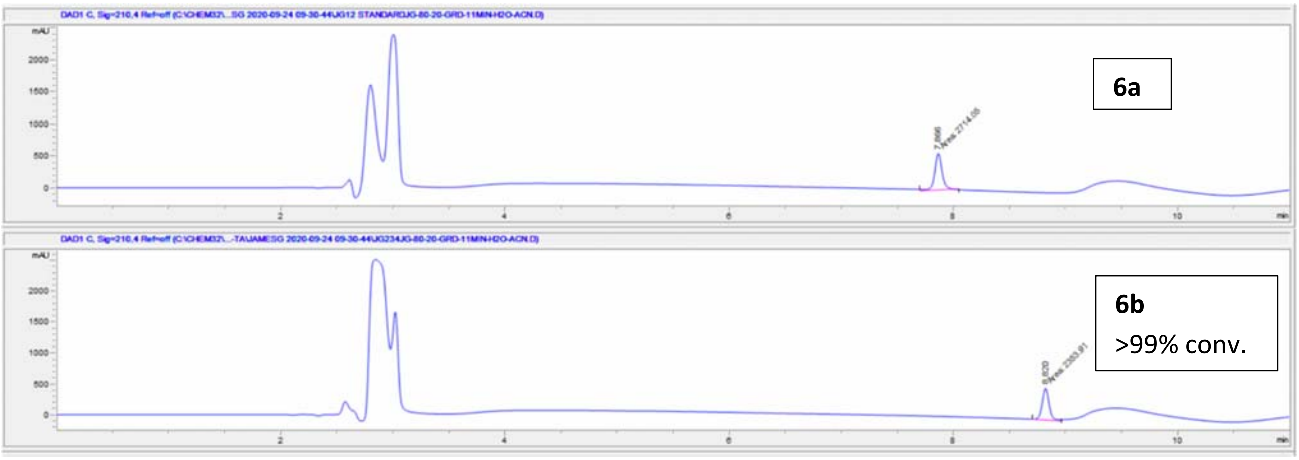
6a
6b
>99% conv.

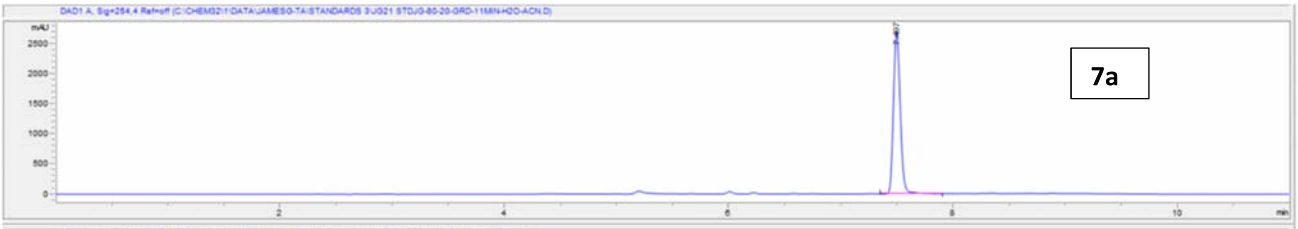
7a

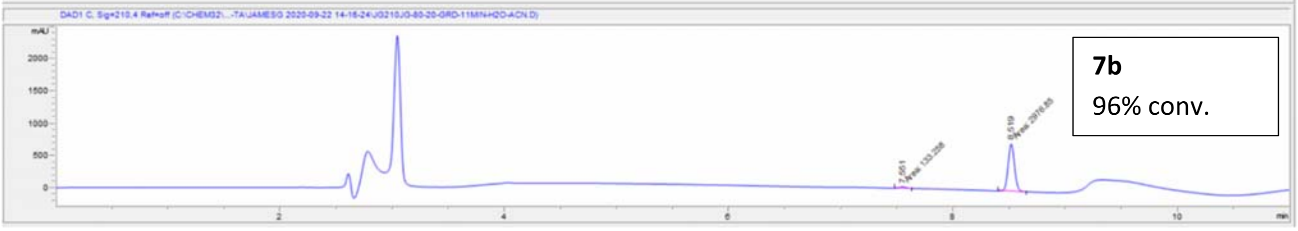
7b
96% conv.

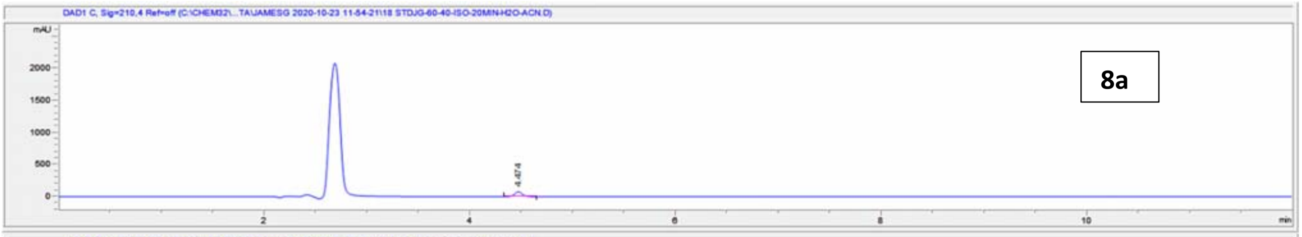
8a

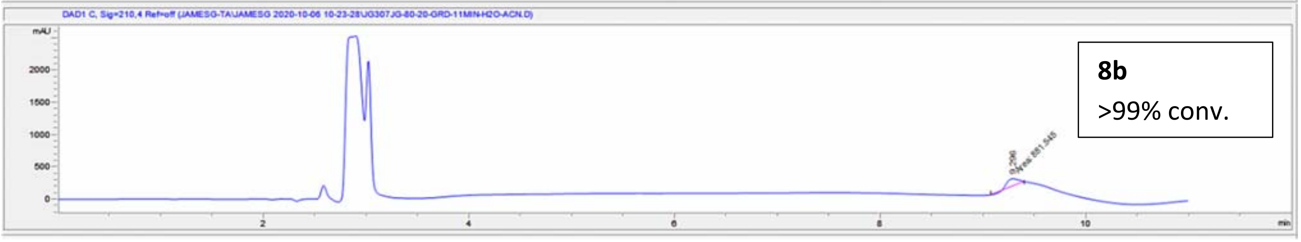
8b
>99% conv.

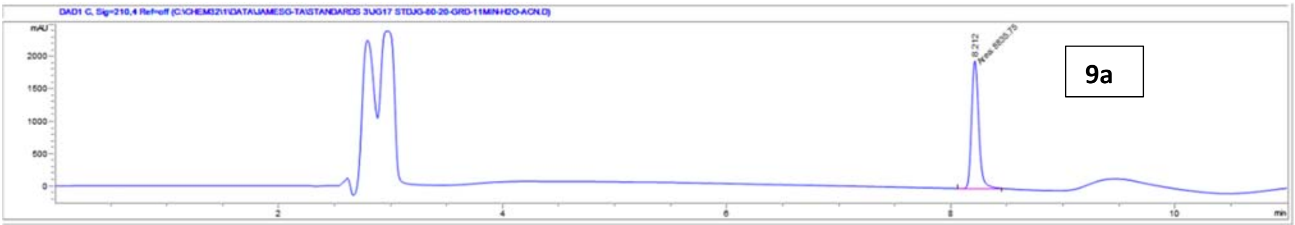
9a

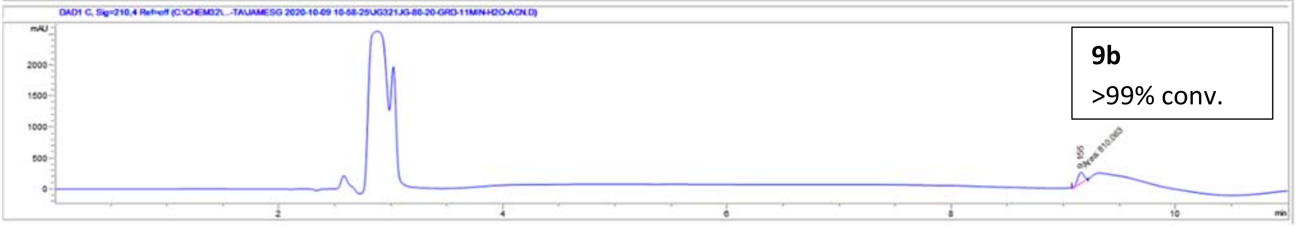
9b
>99% conv.

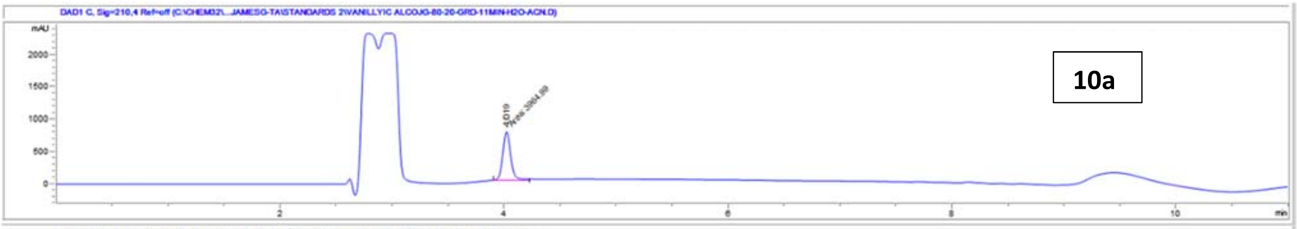
10a

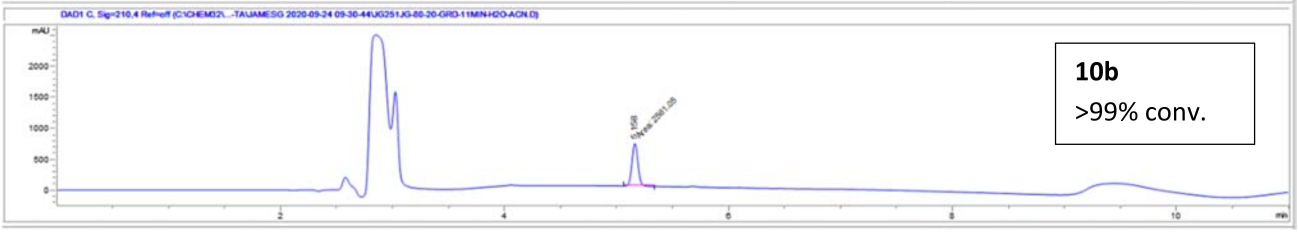
10b
>99% conv.

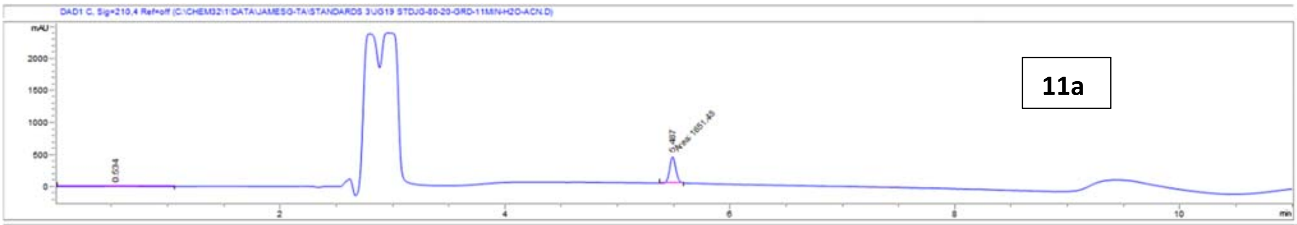
11a

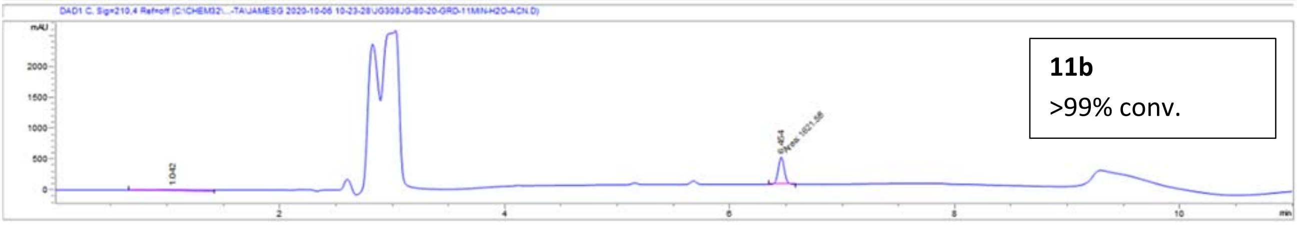
11b
>99% conv.

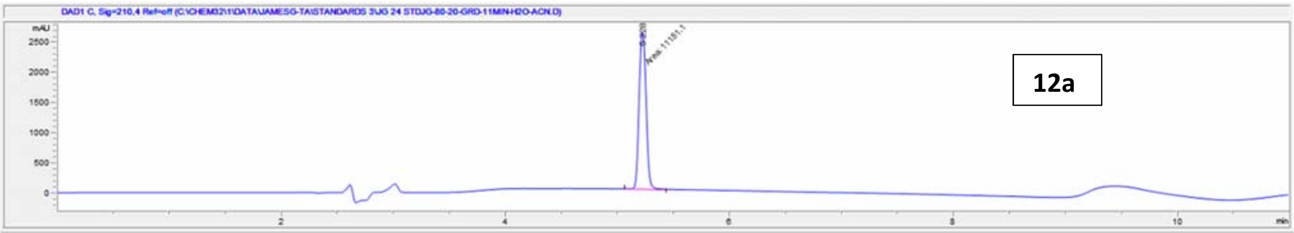
12a

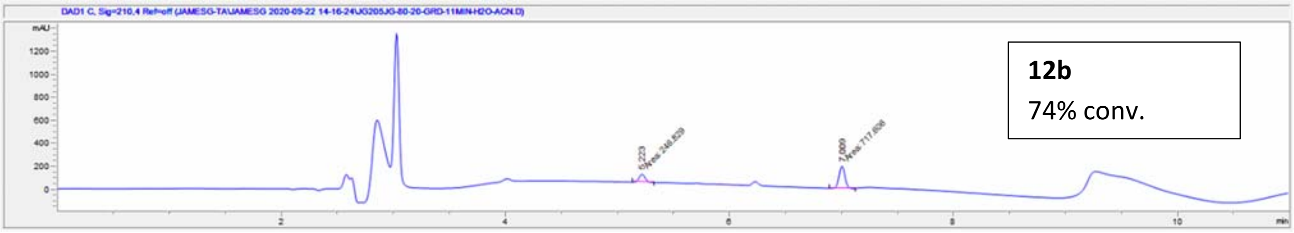
12b
74% conv.

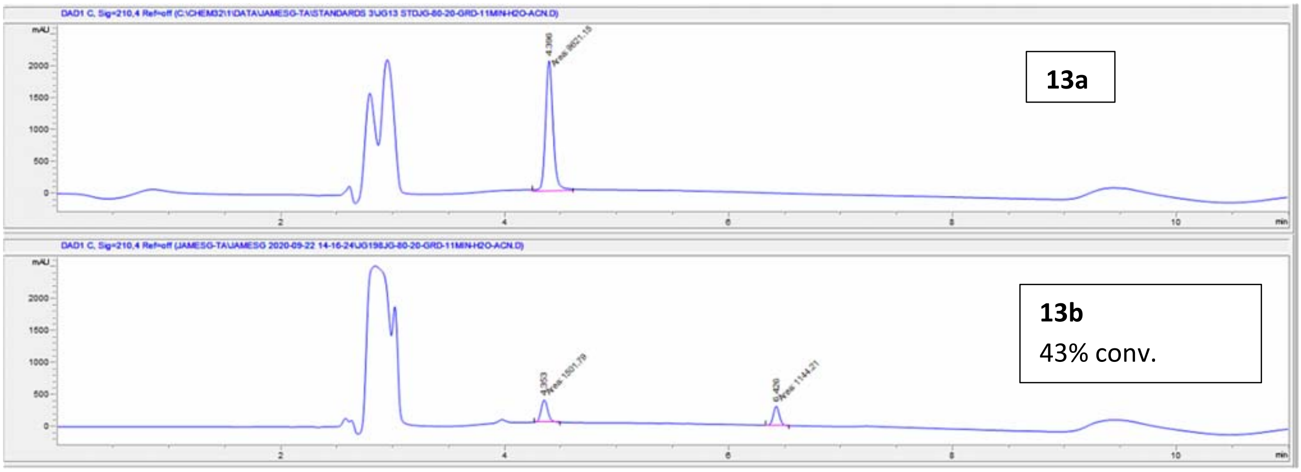
13a
13b
43% conv.

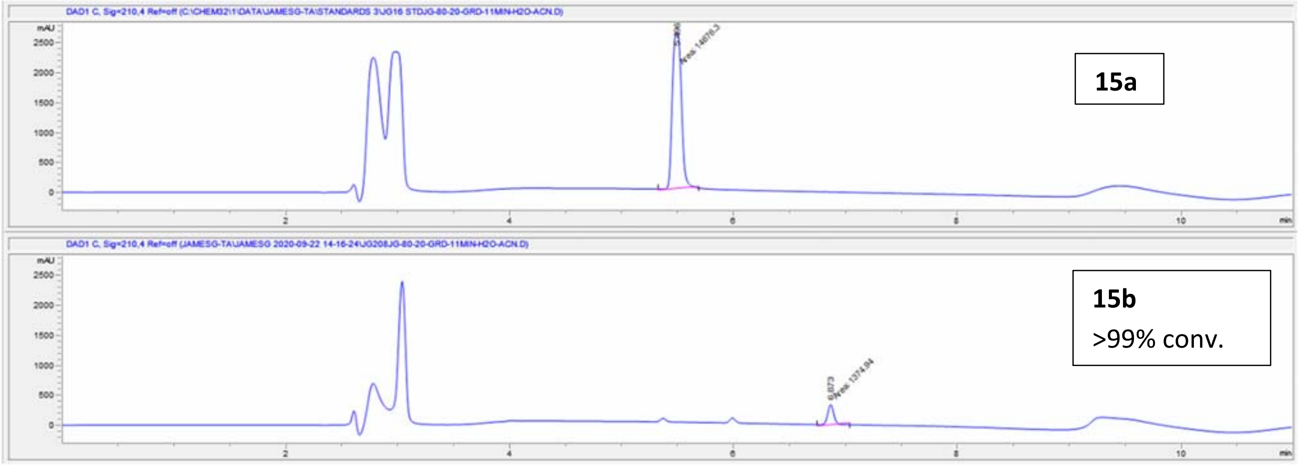
15a
15b
>99% conv.

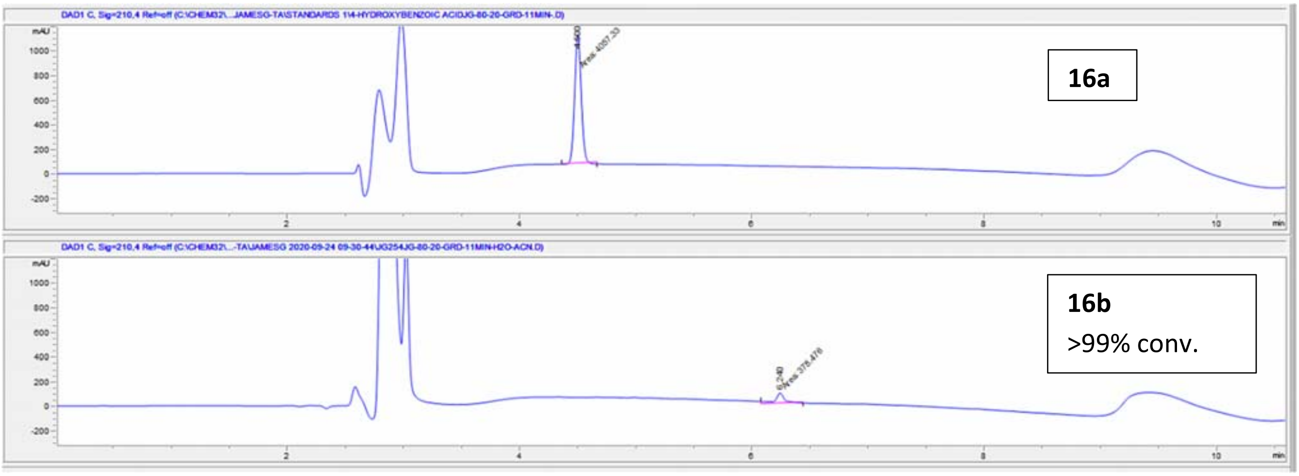
16a
16b
>99% conv.

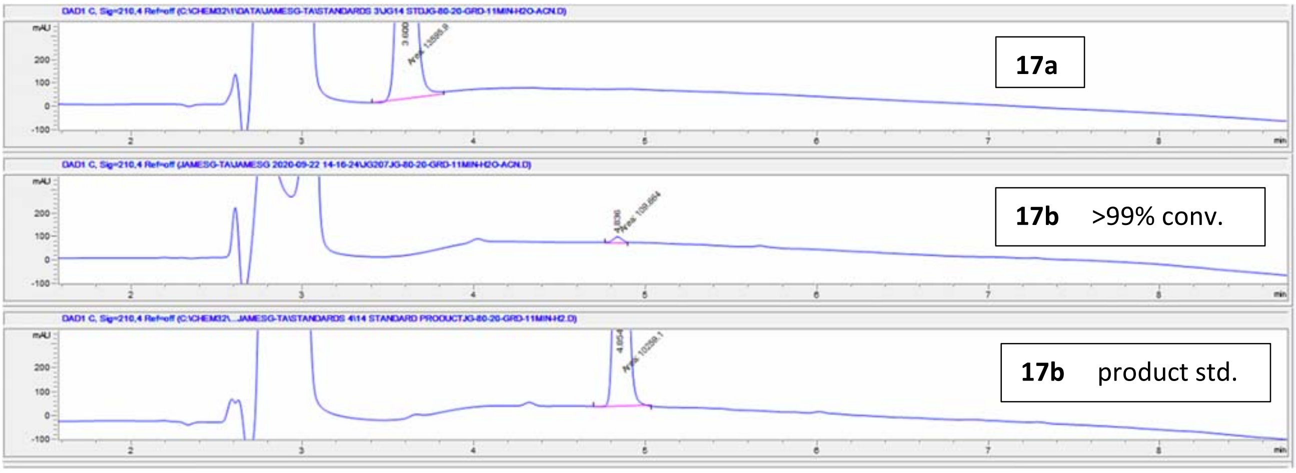
17a
17b >99% conv.
17b product std.

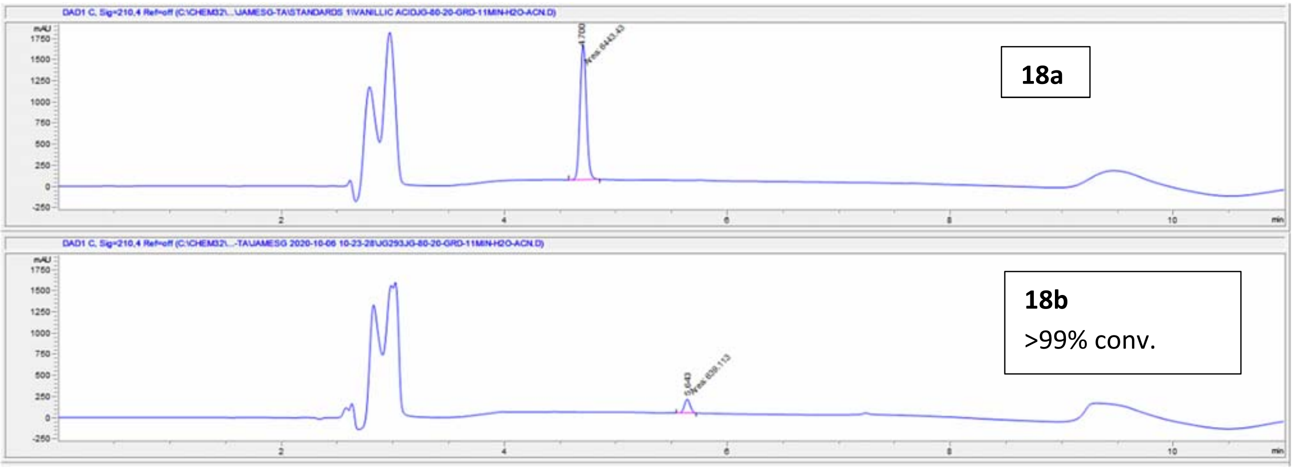
18a
18b
>99% conv.

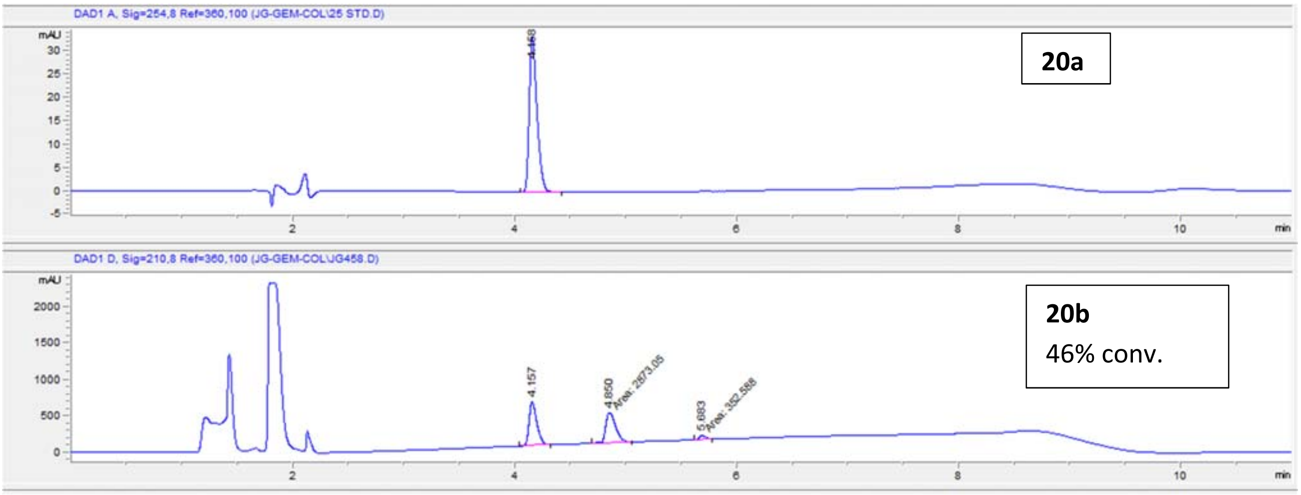
DAD1 A, Sig=254,8 Ref=360,100 (JG-GEM-COL\25 STD.D)
20a
DAD1 D, Sig=210,8 Ref=360,100 (JG-GEM-COL\JG458.D)
20b
46% conv.

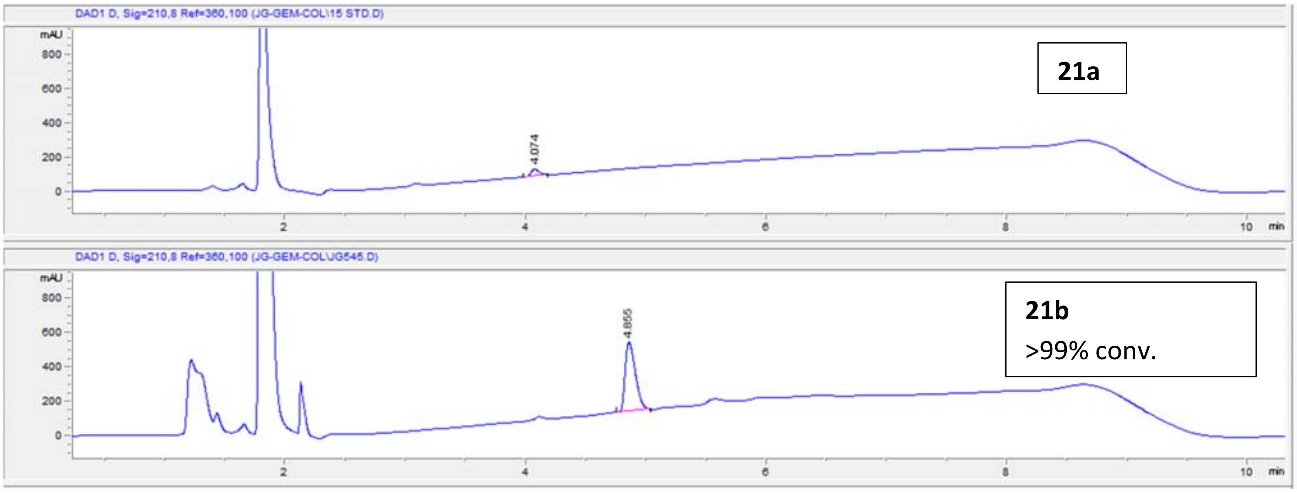
DAD1 D, Sig=210,8 Ref=360,100 (JG-GEM-COL\15 STD.D)
mAU
4.074
min
21a
DAD1 D, Sig=210,8 Ref=360,100 (JG-GEM-COL\JG545.D)
4.855
21b
>99% conv.

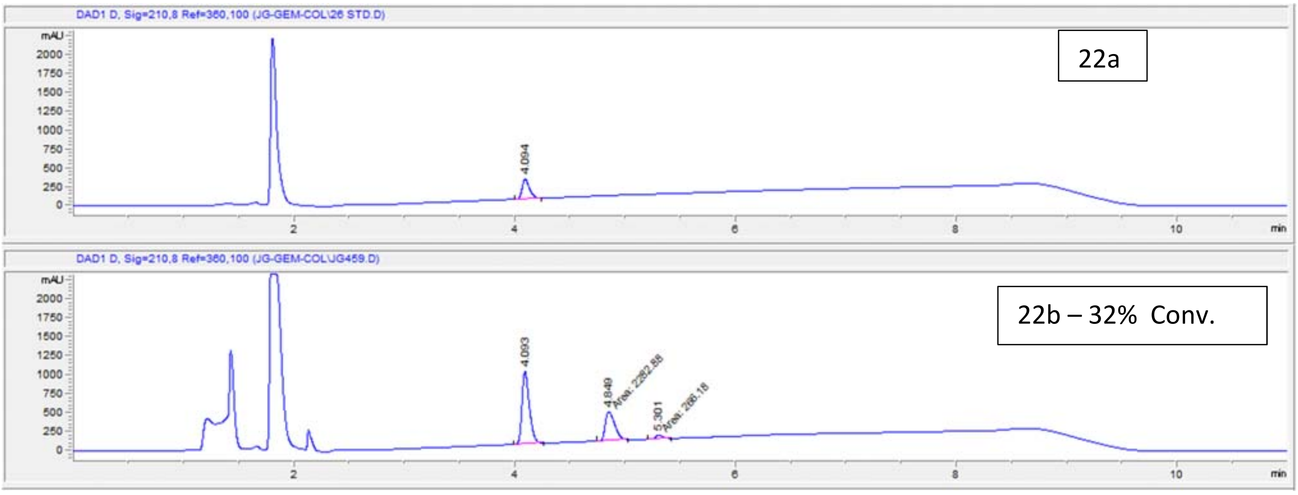
DAD1 D, Sig=210,8 Ref=360,100 (JG-GEM-COL\26 STD.D)
mAU
4.094
min
22a
DAD1 D, Sig=210,8 Ref=360,100 (JG-GEM-COL\JG459.D)
4.093
4.849
Area: 2282.88
5.301
Area: 266.18
22b – 32% Conv.

^{1}H and ^{13}C NMR spectra of isolated L-veratrylglycine

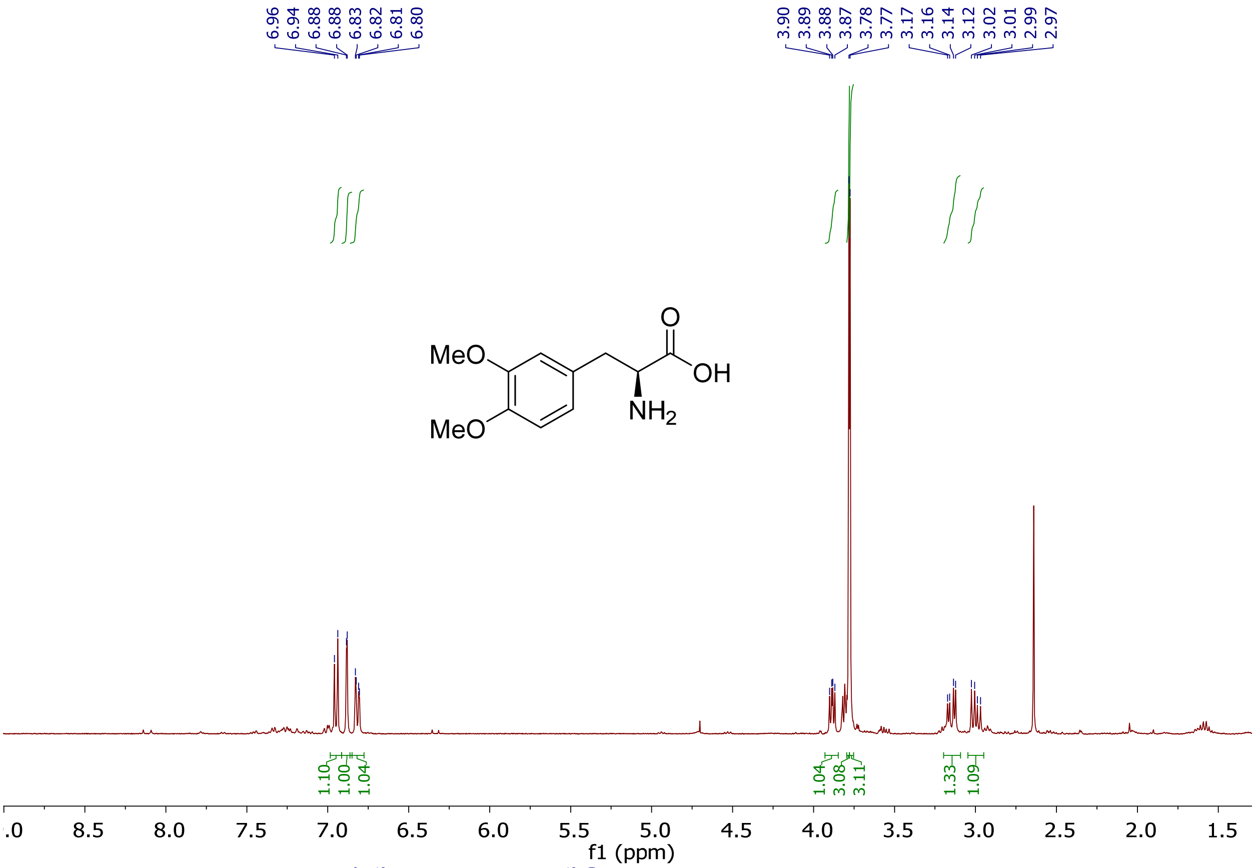

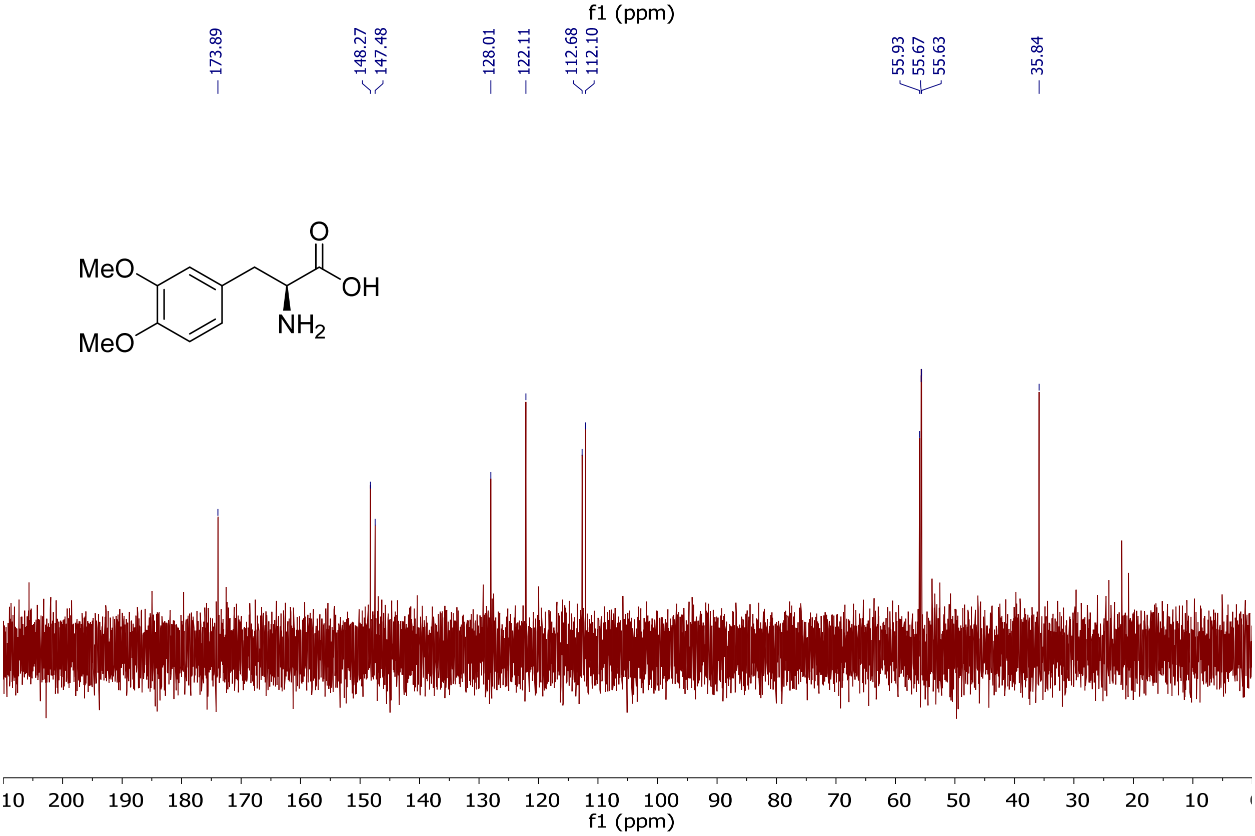

HRMS spectra of isolated L-veratrylglycine

Full spectrum

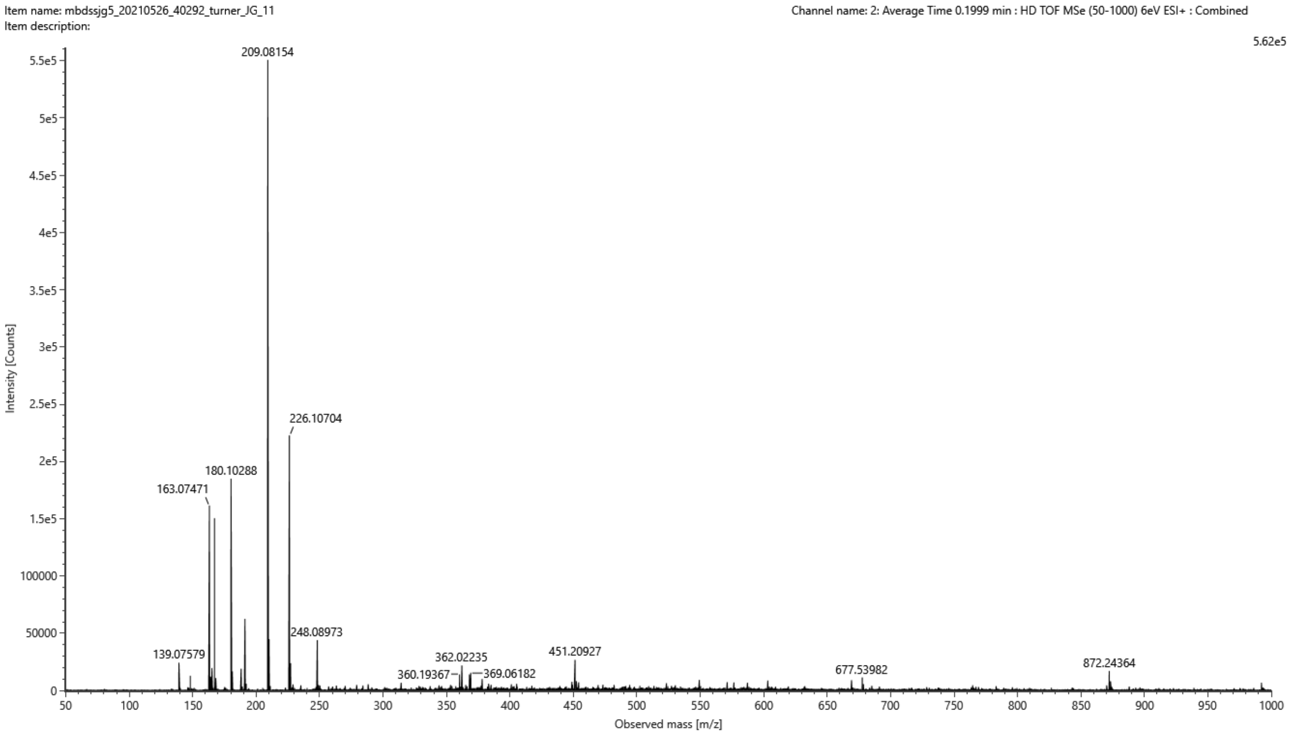

Expansion

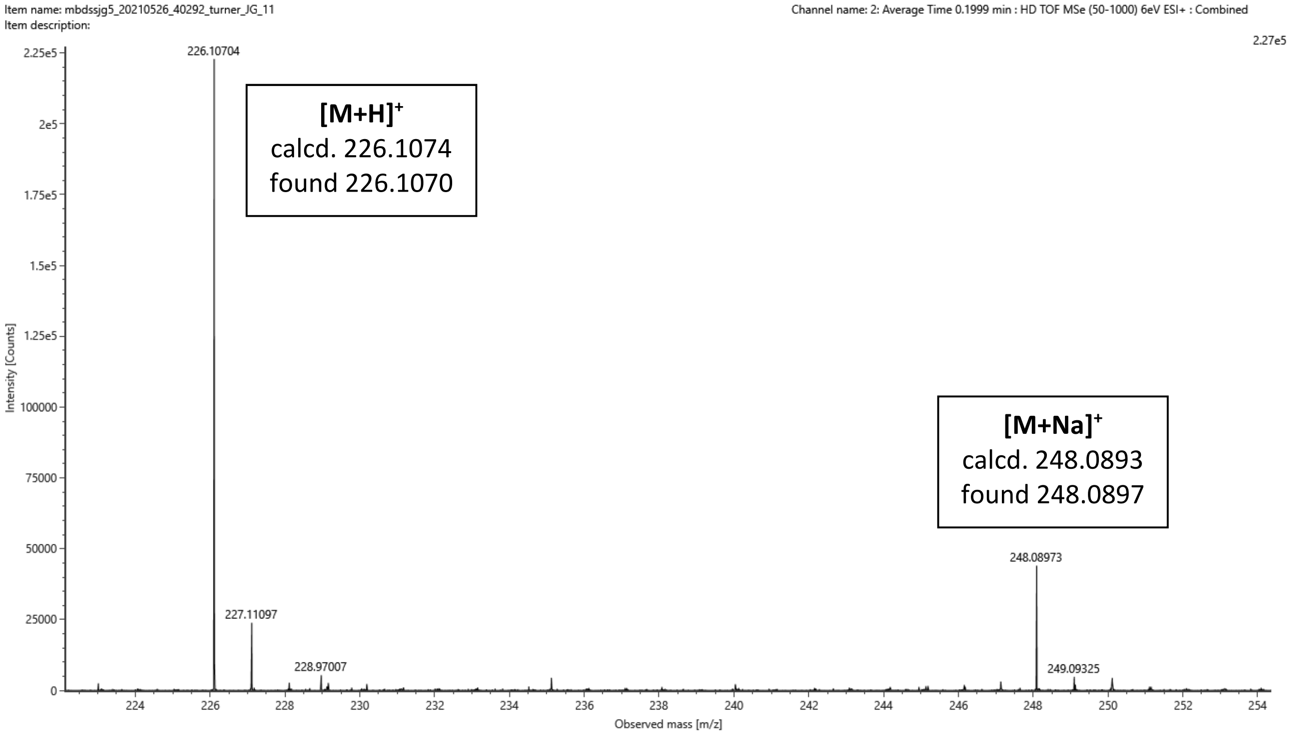

References

(1) Q5® High-Fidelity PCR Kit https://international.neb.com/products/m0492-q5-high-fidelity-2x-master-mix

(2) NEB. NEBuilder HiFi DNA Assembly - The next generation of DNA assembly and cloning https://nebuilder.neb.com/

(3) O. Erster, M. Liscovitch, *Methods Mol. Biol.* **2010**, *634*, 157–174.

(4) H. Liu, J. H. Naismith, *BMC Biotechnol.* **2008**, *8*, 91.

(5) K. A. Datsenko, B. L. Wanner, *Proc. Natl. Acad. Sci. U. S. A.* **2000**, *97*, 6640–6645.

(6) M. F. Gonzales, T. Brooks, S. U. Pukatzki, D. Provenzano, *J. Vis. Exp.* **2013**, *80*, e50684.
